# Simultaneous Detection of Both GDNF and GFRα1 Expression Patterns in the Mouse Central Nervous System

**DOI:** 10.3389/fnana.2016.00073

**Published:** 2016-06-24

**Authors:** Clara Ortega-de San Luis, Alberto Pascual

**Affiliations:** Instituto de Biomedicina de Sevilla (IBiS), Hospital Universitario Virgen del Rocío/CSIC/Universidad de SevillaSeville, Spain

**Keywords:** trophic factors, GDNF, GFRα1, brain connectivity, Parkinson’s disease, addiction-related disorders, motor neurons

## Abstract

Glial cell line-derived neurotrophic factor (GDNF) is proposed as a therapeutic tool in Parkinson’s disease, addiction-related disorders, and neurodegenerative conditions affecting motor neurons (MNs). Despite the high amount of work about GDNF therapeutic application, the neuronal circuits requiring GDNF trophic support in the brain and spinal cord (SC) are poorly characterized. Here, we defined GDNF and GDNF family receptor-α 1 (GFRα1) expression pattern in the brain and SC of newborn and adult mice. We performed systematic and simultaneous detection of EGFP and LacZ expressing alleles in reporter mice and asked whether modifications of this signaling pathway lead to a significant central nervous system (CNS) alteration. GFRα1 was predominantly expressed by neurons but also by an unexpected population of non-neuronal cells. GFRα1 expression pattern was wider in neonatal than in adult CNS and GDNF expression was restricted in comparison with GFRα1 at both developmental time points. The use of confocal microscopy to imaging X-gal deposits and EGFP allowed us to identify regions containing cells that expressed both proteins and to discriminate between auto and non-autotrophic signaling. We also suggested long-range GDNF-GFRα1 circuits taking advantage of the ability of the EGFP genetically encoded reporter to label long distance projecting axons. The complete elimination of either the ligand or the receptor during development did not produce major abnormalities, suggesting a preponderant role for GDNF signaling during adulthood. In the SC, our results pointed to local modulatory interneurons as the main target of GDNF produced by Clarke’s column (CC) cells. Our work increases the understanding on how GDNF signals in the CNS and establish a crucial framework for posterior studies addressing either the biological role of GDNF or the optimization of trophic factor-based therapies.

## Introduction

The glial cell line-derived neurotrophic factor (GDNF) belongs to a group of extracellular ligands distantly related to the transforming growth factor superfamily (Airaksinen and Saarma, 2002). GDNF signals preferentially through extracellular GPI-anchored receptors, GDNF family receptors-α (GFRα1–4), having stronger binding activity over GFRα1 (Trupp et al., 1998). GDNF–GFRα1 complexes recruit either the tyrosine kinase transmembrane protein (RET) or the neural cell adhesion molecule NCAM to initiate intracellular signaling (Durbec et al., 1996; Jing et al., 1996; Trupp et al., 1996; Paratcha et al., 2003). Additionally to the GFRα1 mediated responses, GDNF can directly interact with either integrins or syndecan (Pascual et al., 2011), although this signaling pathway has a smaller contribution. From a functional point of view, GDNF was originally identified as a potent trophic agent that promotes differentiation and survival of dopaminergic neurons (Lin et al., 1993), the main neuronal population affected in Parkinson’s disease (de Lau and Breteler, 2006). In animals, GDNF is required for the maintenance of the nigrostriatal pathway (Pascual et al., 2008) and has been extensively used to ameliorate Parkinson’s disease models (for reviews see Pascual et al., 2011; d’Anglemont de Tassigny et al., 2015; Ibáñez and Andressoo, 2016). In addition to their role in the nigrostriatal pathway, GDNF and GFRα1 are expressed in the dopaminergic mesolimbic circuit (ventral tegmental area—nucleus accumbens; Trupp et al., 1997; Pascual et al., 2008; Hidalgo-Figueroa et al., 2012) where GDNF has a role in survival and circuit functioning (Airavaara et al., 2004; He et al., 2005; Pascual et al., 2008, 2011). Therefore, GDNF was recently proposed as a potential therapy to treat psychostimulant addictions to ethanol and opioids (Carnicella and Ron, 2009). GDNF also has a strong protective effect *in vitro* and *in vivo* over motor neurons (MNs; Henderson et al., 1994; Oppenheim et al., 1995; Bohn, 2004). Adult MNs express GDNF receptors and can bind, internalize and transport the protein in both retro- and anterograde directions (Leitner et al., 1999; Russell et al., 2000). Consequently, GDNF was considered as a potential therapy to treat neurodegenerative disorders associated with the loss of MNs like amyotrophic lateral sclerosis (Bohn, 2004).

Genetically modified mouse models, in which expression of reporter genes are driven either by the endogenous promoter or transcriptional regulatory regions of a particular gene, are a powerful tool to study gene expression patterns (see for instance Gong et al., 2003). Genetic models have several advantages, the reporter signal is stable and heritable and permits, in some cases, the mapping of centimeter-long axons (Feng et al., 2000).

GDNF expression was analyzed in rats by *in situ* hybridization producing contradictory results, probably due to the different sequences used as riboprobes (Trupp et al., 1997; Golden et al., 1999; Barroso-Chinea et al., 2005). In our previous characterization of the brain regions expressing GDNF, we used two genetic engineered mice models. First, *Gdnf^−(LacZ)/+^* mice (Sánchez et al., 1996) allowed for the study of ß-galactosidase (ß-gal) activity as a surrogate marker of the transcriptional activity of the GDNF promoter. The expression pattern observed in *Gdnf*^−(LacZ)/+^ mice closely matched the obtained using *in situ* hybridization (Trupp et al., 1997; Pascual et al., 2008; Hidalgo-Figueroa et al., 2012). The same mouse model was used to map GDNF expression in the spinal cord (SC; Hantman and Jessell, 2010). Second, *Gdnf-Egfp* is a transgenic mouse line generated by the Gene Expression Nervous System Atlas (GENSAT) project at Rockefeller University containing a bacterial artificial chromosome, in which an EGFP coding sequence was inserted after the initial ATG codon of the GDNF sequence. Although this animal model did not fully reproduce the expression pattern observed using *in situ* hybridization or X-gal precipitate detection, an overall agreement was observed (Hidalgo-Figueroa et al., 2012). The expression of GFRα1 has been previously studied using both *in situ* hybridization and immunohistochemistry, but an extensive characterization of the cell types expressing GFRα1 is lacking. Recently, a genetic-modified *Gfr*α*1* allele was described and is available for detailed study. The *Gfr*α*1*^−(Egfp)/+^ reporter mouse carries a *Gfr*α*1* null and *Egfp-*targeted knock-in (KI) allele (Uesaka et al., 2007).

Here we asked whether a combination of reporter mice could enable the simultaneous identification of GDNF and GFRα1 expressing cells. This study has not been previously addressed mainly due to the lack of good antibodies and the low expression levels of both proteins. Until recently, the use of *LacZ* reporter mice for confocal studies was limited by the ability of the anti-ß-gal antibody to detect the reporter protein levels attained by a particular promoter. However, we employed here a recently developed technique that exploits the fluorescence of X-gal deposits (Hidalgo-Figueroa et al., 2012; Levitsky et al., 2013) to perform confocal imaging of EGFP (GFRα1 reporter) and X-gal (GDNF reporter) signals. We also wondered if genetic deletion of GDNF or GFRα1 affects the survival or the maintenance of the identified neuronal populations.

## Materials and Methods

### Animals

Mice were housed under temperature-controlled conditions (22°C) in a 12 h light/dark cycle with access *ad libitum* to food and water. Housing and treatments were performed according to the animal care guidelines of European Community Council (86/609/EEC). The Animal Research Committee at the Hospital Universitario Virgen del Rocio approved all procedures. In every experiment, both sexes were analyzed and no differences were found between them. A description of the mouse lines used in this study is presented in Supplementary Table 1, as well as their corresponding figure.

#### *Gfrα1^Flox^* and *Gfrα1^−(Egfp)^*

**Figure 1:**
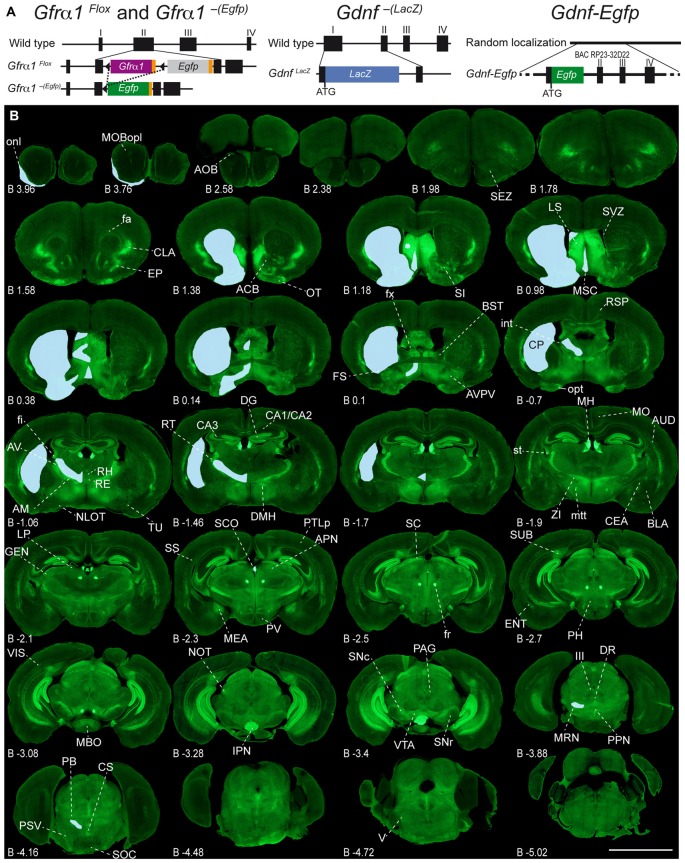
**Adult expression of GDNF family receptor-α 1 (GFRα1) and Glial cell line-derived neurotrophic factor (GDNF). (A)** Schematic drawing of the mice models used in this work. *Gfr*α*1*^Flox^ and *Gfr*α*1*^−(Egfp)^. *Gfr*α*1*^Flox^ allele: functional *Gfr*α*1* cDNA (purple box) flanked by loxP sites (black triangles) disrupts *Gfr*α*1* wild-type allele. Cre-recombinase activity (dotted line) generates *Gfr*α*1*^−(Egfp)^ knock-out (KO) allele and enables *Egfp* expression (green box). *Gdnf*^−(LacZ)^ allele: *LacZ* gene (blue box) fused in frame to *Gdnf* exon I to generate a null allele. *Gdnf-Egfp*: random insertion of bacterial artificial chromosome (BAC RP23-32D22) incorporating *Egfp* gene (green box) in frame with *Gdnf* initial ATG. **(B)** Expression of GFRα1 (anti-EGFP, green) and GDNF (X-gal positive, blue areas drawn over left hemispheres) in 50 μm-thick coronal brain sections from 1-month-old *Gfr*α*1*^−(Egfp)/+^; *Gdnf*^−(LacZ)/+^ mouse. Scale bar represents 5 mm. Distance from bregma (B) is indicated (mm). Abbreviations are listed in Supplementary Table 3.

*Gfr*α*1*^Flox^ mice (a kind gift from J. Milbrandt, Washington University School of Medicine, St. Louis, MO, USA) were generated to conditionally inactivate GDNF receptor and to investigate the physiological function of GDNF signaling in later enteric nervous system development (Uesaka et al., 2007). This mice have been used in the literature to analyze GDNF-GFRα1 signaling and site-specific gene expression (Uesaka et al., 2015; Mwangi et al., 2016). In this line, CRE-mediated recombination transforms the functional *floxed* GFRα1 allele, *Gfr*α*1*^Flox^, into a null EGFP reporter allele, *Gfr*α*1*^−(Egfp)^ (Figure 1A, left). Here we generated the *Gfr*α*1*^−(Egfp)^ allele by crossing *Gfr*α*1*^Flox/+^ mice with *Th-IRES-Cre* mice (Supplementary Table 1; Lindeberg et al., 2004). The *Th-IRES-Cre* line showed Cre recombinase activity in the germline (Díaz-Castro et al., 2012), generating an ubicuous *Gfr*α*1*^−(Egfp)^ non-functional allele that expressed *Egfp*. Full deletion of the receptor was generated by crossing *Gfr*α*1*^−(Egfp)/+^ mice and selecting both GFRα1 KO (*Gfr*α*1*^−(Egfp)/-(Egfp)^) and control mice (*Gfr*α*1*^−(Egfp)/+^; Supplementary Table 1).

#### *Gdnf*^−(LacZ)/+^

To analyze GDNF expression, we used *Gdnf*^−(LacZ)/+^ mice where *LacZ* gene is fused in frame to *Gdnf* exon I generating a null reporter allele (Sánchez et al., 1996; Figure 1A, center). This mice have been extensively used and validated in function and expression analysis (Villadiego et al., 2005; Pascual et al., 2008; Hidalgo-Figueroa et al., 2012; Muñoz-Bravo et al., 2013). To simultaneously analyze *Gdnf* and *Gfr*α*1* expression patterns, we crossed *Gdnf*^−(LacZ)/+^ with *Gfr*α*1*^−(Egfp)/+^ mice to generate the double heterozygous (*Gdnf*^−(LacZ)/+^; *Gfr*α*1*^−(Egfp)/+^; Supplementary Table 1). Full deletion of *Gdnf* was obtained in mice that also carry a copy of the *Gfr*α*1*^−(Egfp)^ allele by crossing *Gfr*α*1*^−(Egfp)/+^; *Gdnf*^−(LacZ)/+^ with *Gdnf*^−(LacZ)/+^; *Gfr*α*1*^+/+^ mice. From this cross, we selected GDNF KO (*Gfr*α*1*^−(Egfp)/+^; *Gdnf*^−(LacZ)/-(LacZ)^) as well as control (*Gfr*α*1*^−(Egfp)/+^; *Gdnf*^−(LacZ)/+^) mice (Supplementary Table 1).

#### Gdnf-Egfp

To analyze projections from neurons expressing *Gdnf*, transgenic *Gdnf-Egfp* mice were obtained from GENSAT (RRID: nif-0000-00130) on a mixed background (Gong et al., 2003). This line was generated by random insertion of a bacterial artificial chromosome containing regulatory sequences of *Gdnf* expression followed by *Egfp* reporter gene (Figure 1A, right; Supplementary Table 1). *Gdnf-Egfp* mice have been previously validated for the analysis of *Gdnf* expression (Hidalgo-Figueroa et al., 2012).

#### Emx1-Cre

We used *Emx1-Cre* mice (Gorski et al., 2002; RRID: IMSR_JAX:005628) to check whether dorsal corticospinal pathway cells, Emx1+, express GFRα1. First, we validated recombination in these cells by crossing with recombination-marker *R26R-YFP* line (RRID: IMSR_006148; Supplementary Table 1). Secondly, we crossed *Emx1-Cre* line with *Gfrα1^Flox/+^* mice, to generate *Emx1^Cre/+^*; *Gfrα1^Flox/+^* mice (Supplementary Table 1).

### Histological Analyses

#### Tissue Preparation

To analyze brain expression, adult mice (postnatal day 30–90, P30–P90) were anesthetized with intraperitoneal injection of tiobarbital (Braun), and transcardially perfused with phosphate-buffered saline (PBS) followed by 4% Paraformaldehyde (PFA, Sigma) in PBS. Brains were removed immediately, post-fixed overnight at 4°C with the same fixative, included in gelatin (Panreac) and cut in 50 μm thick coronal sections using a vibratome (Leica). Preparation of neonatal brains (postnatal day 0–1, P0–P1) was similar, avoiding the initial perfusion step. SC was removed from anesthetized mice and fixed overnight (15 h) at 4°C with 4% PFA in PBS. Tissue was incubated in Sucrose 30% PBS overnight, included in OCT (Sakura) medium and cut in 20 μm thick coronal cryostat sections (Leica).

#### Immunohistochemistry

For simultaneous histological detection of GDNF and GFRα1 expression in *Gfr*α*1*^−(Egfp)/+^; *Gdnf*^−(LacZ)/+^ mice, the 5-bromo-4-chloro-3-indolyl-D-galactopyranoside (X-gal) staining was performed on every sixth section (adult mice) or every fourth (neonatal mice). Sections were washed three times in Solution C (MgCl_2_ 2 mM, Sigma; Igepal 0.02%, Fluka; Na deoxycholate 0.01%, Sigma; EGTA 5 mM, Sigma; in PBS, pH 7.4) and incubated overnight in staining solution (K_3_FeCN_6_ 10 mM, Sigma; K_4_FeCN_6_ 10 mM, Sigma; 0.5 mg/ml X-gal, Sigma; resuspended in N-N dimethylformamide, Sigma; in solution C). This reaction was followed by an immunohistochemistry against green fluorescent protein (antibody anti-GFP, 1:1000; Molecular Probes Invitrogen; Cat# A11122; RRID: AB_2307355). For immunohistochemistry reaction, tissue was permeabilized with 0.3% Triton X-100 (Sigma) in PBS for 10 min, washed twice with 0.1% Triton X-100 in PBS, blocked in Gelatin 0.02% solution in 0.25% Triton X-100 in PBS for 1 h and incubated overnight at 4°C with primary antibodies in blocking solution (information about antibody characterization and working dilution in Supplementary Table 2). After three washes with 0.1% Triton X-100 in PBS, slices were incubated with secondary antibody anti-rabbit IgG conjugated with Alexa-488 (1:800; Life Technologies, Cat# A11034; RRID: AB_10562715) and/or anti-mouse IgG conjugated with Alexa-568 (1:800, Life Technologies; Cat# A11031; RRID: AB_10562420). After two washes with PBS, nuclei were stained with DAPI (Sigma, 1:1000). The sections were mounted onto SuperFrost slides (Fisher Scientific), dried and cover slipped with Dako fluorescent mounting medium (Dako).

#### Antibody Specificity

*Anti-GFP* (1:1000; Molecular Probes Invitrogen; Cat# A11122 RRID: AB_2307355) is a rabbit polyclonal antibody (IgG fraction) obtained after the immunization with GFP isolated from *Aequorae victoria*. The antibody did not stain sections from littermates that had not undergone Cre recombination, therefore lacking EGFP expression. Moreover, the expression pattern of GFRα1 analyzed by immunohistochemistry in *Gfr*α*1*^−(Egfp)/+^, as well as *Gfr*α*1*^−(Egfp)/+^; *Gdnf*^−(LacZ)/+^ mice was similar to previous studies (Trupp et al., 1997; Golden et al., 1999).

*Anti-BrdU* (1:200; rat, Abcam, Cat# AB6326; RRID: AB_305426) is a rat monoclonal antibody that detects nucleated cells in S-Phase that have incorporated BrdU, as a thymidine analog, into their DNA. The specificity of the antibody was tested by immunohistochemistry on SC from mice descendent from females not injected with BrdU during pregnancy.

*Anti-NeuN* (1:500, mouse, Merck Cat# MAB377 RRID: AB_11210778) is a mouse monoclonal antibody against a neuron-specific nuclear protein (Neuronal Nuclei, NeuN). It has been probed specific as a neuronal marker which labels neuronal nuclei in a pattern similar to published (Mullen et al., 1992).

### Imaging and Data Analysis

#### Low Magnification Analysis

First, we imaged whole brain sections with a fluorescence and light microscope (objective 40×/0.95, Olympus BX-61) and the Superimage composition tool included in the NewCAST Software package (Visiopharm). Superimages obtained with this system were edited in Adobe Photoshop CS5 Software in order to adjust brightness, contrast and sharpness. Second, by visual microscopy analysis (40×/0.95, Olympus BX-61 and NewCAST, Visiopharm) we identified X-gal precipitates in bright field and locate their position in the brain, in at least three mice by age for every structure. Using Adobe Photoshop CS5, we manually added a blue area to the Superimage in the same area where the signal was detected by bright field microscopy. Then, anatomical identification of brain structures was done by comparison with Allen Reference Atlas (RRID: nlx_21010; Lein et al., 2007) for adult mice and with the Atlas of the developing mouse brain for neonatal animals (Paxinos et al., 2007). High-resolution figures are available under request.

#### High Magnification Analysis

GDNF and GFRα1-positive areas were later imaged at high magnification using a confocal microscope (Objective plan APO 60×/1.40 Oil, Nikon A1R+) in Z-Stacks compositions of 10–20 optical planes of 1 μm thickness. Using both visual microscopy analysis and image analysis, we classified expression as neuronal (identified by NeuN positive marker), cellular (NeuN negative) or axonal (identified by morphology using high magnification analysis). Every brain structure was classified by analysis of at least three mice by age.

#### Colocalization Confocal Analysis

Confocal images to probe colocalization of X-gal precipitates and GFP fluorescent signal were acquired using a Leica TCS SP2 microscope, as described (Levitsky et al., 2013). This technique, based on X-gal fluorescence emission and mathematical optical correction, allows to directly image X-gal staining on thick tissue sections by confocal microscopy. As there is not need to use specific antibodies against ß-gal, this technique has lower detection threshold, high specificity and reliability. Its use has been previously validated for the detection of GDNF-positive cells (Hidalgo-Figueroa et al., 2012). Using this technique, we systematically analyzed colocalization of X-gal perinuclear precipitates with anti-EGFP inmunofluorescent signal in at least three mice.

### BrdU Tracing and Quantification

At chosen embryonic development days (from embryonic day 9–15, E9–E15), a single intraperitoneal injection of BrdU (Sigma, 15 mg/mL in PBS and 50 mg/kg of body weight) was performed in pregnant *Gdnf-Egfp* mice derived from timed mating. Pumps were sacrificed at P7, and immunostaining anti-GFP in every fourth section of SC was performed as explained. Immediately after, samples were post-fixated with 4% PFA for 1 h and treated with 2 M HCl for 20 min to denature DNA, followed by incubation with 0.1 M sodium borate, pH 8.5 for 10 min to neutralize HCl. Sections were subjected to heat-induced antigen retrieval in sodium citrate buffer 0.01 M, pH 6.0 at 95°C. After that, the standard protocol described above was performed, using an anti-BrdU primary antibody (1:200, rat, Abcam Cat# AB6326, RRID: AB_305426) and an anti-IgG rat conjugated to Alexa-546 as a secondary antibody (1:500, Life Technologies Cat# A11081 RRID: AB_10563603). Quantification of GDNF+ cells in SC was done manually, by counting all the positive cells on every fourth section (20 μm) from second vertebrae at thoracic (T2) to first at sacral level (S1). The SC from three mice of every age were quantified.

## Results

### Experimental Models

To characterize the expression sites of both GFRα1 and GDNF, we employed three well-characterized genetic models (Figure 1A, Supplementary Table 1 and see “Materials and Methods” Section). GFRα1 expression was followed using a heterozygous allele where the wild-type sequence has been substituted by an *Egfp* (*Gfr*α*1*^−(Egfp)/+^, Uesaka et al., 2007). GDNF expression was analyzed using both a heterozygous KI allele, where the *Gdnf* promoter drove the expression of a *LacZ* gene (*Gdnf*^−(LacZ)/+^, Sánchez et al., 1996), and a heterozygous random insertion of a bacterial artificial chromosome containing the *Gdnf* genomic region, where the coding sequence has been replaced by an *Egfp* (*Gdnf-Egfp*, Gong et al., 2003).

### Adult Brain Regions Expressing GDNF and GFRα1

**Figure 2:**
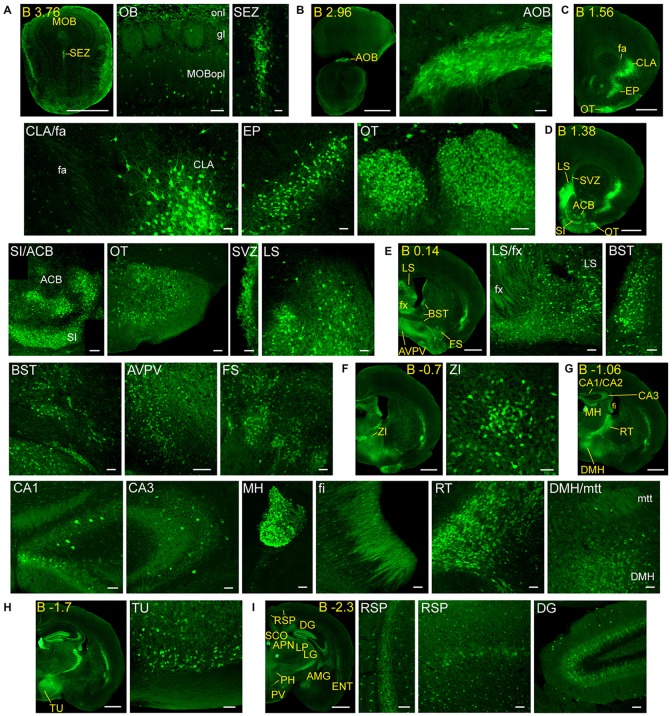
**Detail of anterior GFRα1-positive areas in the adult *Gfr*α*1*^−(*Egfp*)/+^; *Gdnf*^−(*LacZ*)/+^ mouse brain. (A–I)** Coronal brain sections immunostained with an anti-EGFP antibody (green). Yellow-labeled panels: low magnification images indicating the position of confocal images. White-labeled panels: confocal images, Z-stacks projections of 10–20 optical planes (1 μm thickness). Scale bars represent 1 mm in yellow-labeled panels and 50 μm in white-labeled panels. Abbreviations are listed in Supplementary Table 3, except for gl, glomerular layer. Images are oriented so that up is dorsal, and midline is in the left part of the panels. Distance from bregma (B) is indicated (mm) in low magnification images.

**Figure 3:**
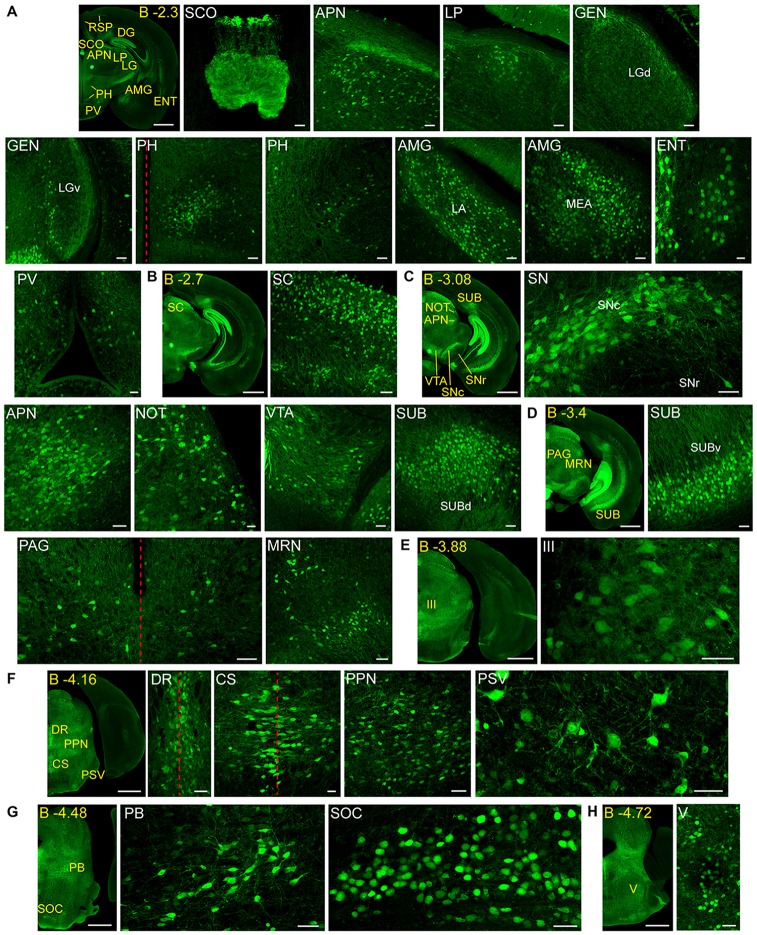
**Detail of posterior GFRα1-positive areas in the *Gfr*α*1*^−(*Egfp*)/+^; *Gdnf*^−(*LacZ*)/+^ mouse brain. (A–H)** Coronal brain sections immunostained with an anti-EGFP antibody (green). Yellow-labeled panels: low magnification images indicating the position of confocal images. White-labeled panels: confocal images, Z-stacks projections of 10–20 optical planes (1 μm thickness). Scale bars represent 1 mm in yellow-labeled panels and 50 μm in white-labeled panels. Abbreviations are listed in Supplementary Table 3, except for LGd and LGv, dorsal (d) and ventral (v) parts of the lateral geniculate complex; LA, lateral amygdalar nucleus; MEA, medial amygdalar nucleus; and SUBd and SUBv, dorsal (d) and ventral (v) parts of subiculum (SUB). Red dotted line in **(A,D,F)** panels indicate midline. Images are oriented so that up is dorsal, and midline is in the left part of the panels. Distance from bregma (B) is indicated (mm) in low magnification images.

**Figure 4:**
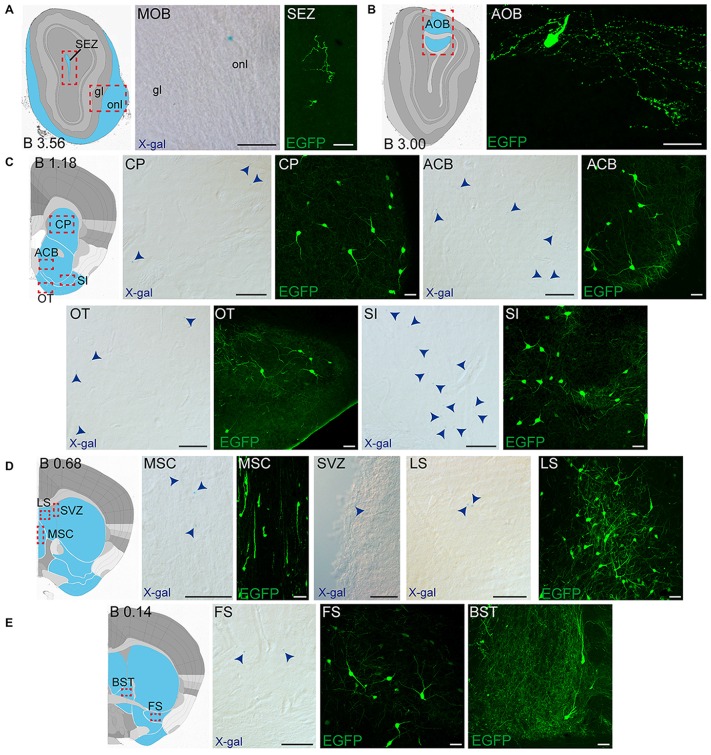
**Detail of anterior GDNF-positive areas in the adult *Gfr*α*1*^−(*Egfp*)/+^; *Gdnf*^−(*LacZ*)/+^ and *Gdnf-Egfp* mouse brains. (A–E)** Modified schemes from Allen Brain Atlas indicate localization of high-magnified pictures (red squares) and GDNF-positive areas (blue). Distance from bregma (B) is noted (mm). Bright field images from *Gfr*α*1*^−(Egfp)/+^; *Gdnf*^−(LacZ)/+^ show X-gal deposits (blue arrowheads when required). Confocal images (Z-stacks projections of 10–20 optical planes of 1 μm thickness) from *Gdnf-Egfp* (anti-EGFP, green). Scale bars indicate 50 μm. Abbreviations are listed in Supplementary Table 3. Images are oriented so that up is dorsal, and midline is in the left part of the panels.

**Figure 5:**
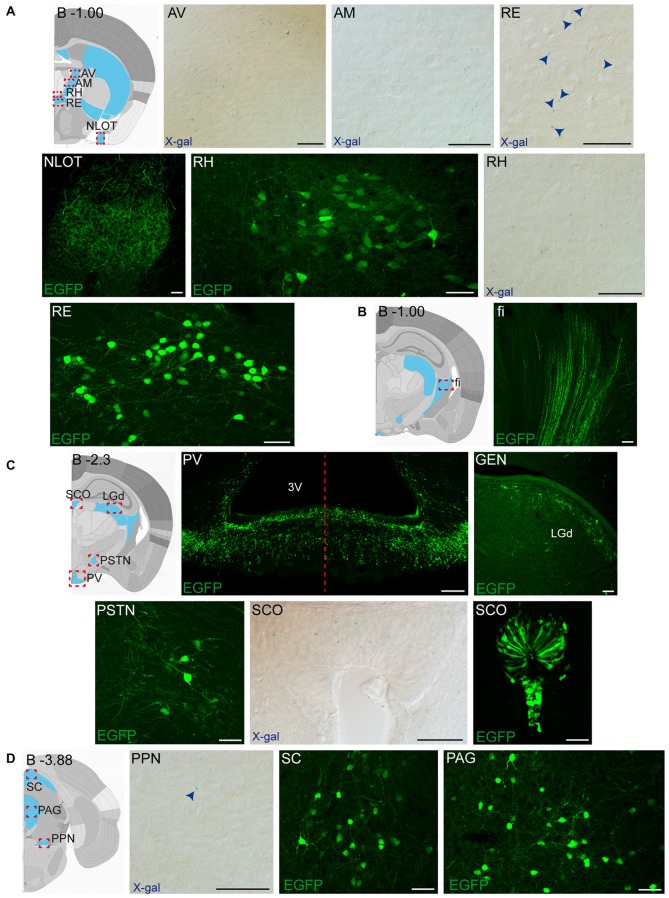
**Detail of posterior GDNF-positive areas in the adult *Gfr*α*1*^−(*Egfp*)/+^; *Gdnf*^−(*LacZ*)/+^and *Gdnf-Egfp* mouse brains. (A–D)** Modified schemes from Allen Brain Atlas indicate localization of high-magnified pictures (red squares) and GDNF-positive areas (blue). Distance from bregma (B) is noted (mm). Bright field images from *Gfr*α*1*^−(Egfp)/+^; *Gdnf*^−(LacZ)/+^ show X-gal deposits (blue arrowheads when required). Confocal images (Z-stacks projections of 10–20 optical planes of 1 μm thickness) from *Gdnf-Egfp* (anti-EGFP, green). Scale bars indicate 50 μm. Abbreviations are listed in Supplementary Table 3. Images are oriented so that up is dorsal, and midline is in the left part of the panels. Red dotted line in **(C)** indicates midline.

In order to gain an understanding of the adult expression pattern of GFRα1 and GDNF, we described the expression of EGFP and ß-gal in *Gfrα1^−(Egfp)/+^*; *Gdnf*^−(LacZ)/+^ mice. Areas with expression of both proteins were characterized, were depicted in Figure 1B and listed in Supplementary Table 3 according to Allen Brain Atlas categorization. Cells were classified as neurons when immunohistochemistry against NeuN showed colocalization with the anti-EGFP signal in *Gfr*α*1*^−(Egfp)/+^; *Gdnf*^−(LacZ)/+^ brain sections (data not shown). The number of cells with GDNF or GFRα1 expression in a particular structure was represented with a semi-quantitative scale in Supplementary Table 3. Regions with neuronal projections were also shown in Supplementary Table 3 and, again, a semi-quantitative scale was used to classify the intensity of the expression. To put our finding in context, we also described if the identified area with either GDNF or GFRα1 expression was already described in the literature or is a new finding of this study (Supplementary Table 3). Areas with GFRα1 expression were imaged at high-magnification using confocal microscopy (Figures 2, 3) and details of the GDNF expression in *Gfr*α*1*^−(Egfp)/+^; *Gdnf*^−(LacZ)/+^ and *Gdnf-Egfp* brains can be observed at high resolution in Figures 4, 5. The GDNF expressing areas observed in the *Gdnf*^−(LacZ)/+^ mouse were confirmed in brain coronal sections from *Gdnf-Egfp* animals (Supplementary Figure 1). To further confirm the identity of the brain nuclei with GFRα1 and GDNF expression, we also analyzed sagittal sections from *Gfr*α*1*^−(Egfp)/+^ and *Gdnf-Egfp* mice (data not shown and Supplementary Table 3).

### Neonatal Brain Regions Expressing GDNF and GFRα1

**Figure 6:**
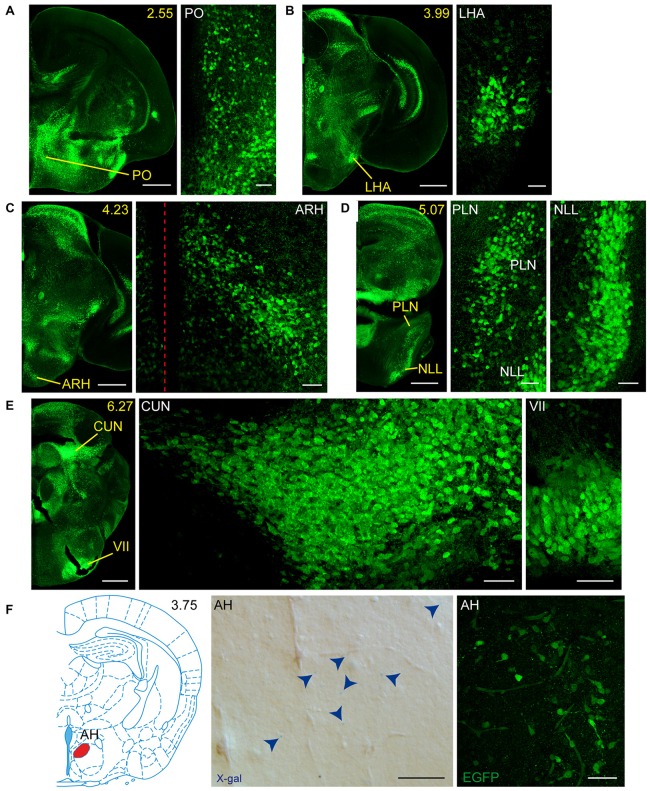
**Detail of areas GFRα1-positive and GDNF-positive in the perinatal but not adult mouse brains. (A–E)** Detail of GFRα1-positive areas in coronal brain sections from newborn *Gfr*α*1*^−(Egfp)/+^; *Gdnf*^−(LacZ)/+^ mice immunostained with an anti-EGFP antibody (green). Yellow-labeled panels: low magnification images indicating the position of confocal images. White-labeled panels: confocal images, Z-stacks projections of 10–20 optical planes (1 μm thickness). Scale bars represent 500 μm in yellow-labeled panels and 50 μm in white-labeled panels. **(F)** Detail of GDNF-positive area in the brain of neonatal *Gfr*α*1*^−(Egfp)/+^; *Gdnf*^−(LacZ)/+^ and *Gdnf-Egfp* mice. Left: modified atlas scheme (Paxinos et al., 2007) indicates localization of high-magnified pictures (in red). Center: bright field image from *Gfr*α*1*^−(Egfp)/+^; *Gdnf*^−(LacZ)/+^ (X-gal positive, blue arrowheads). Right: confocal image (Z-stacks projection of 10–20 optical planes of 1 μm thickness) from *Gdnf-Egfp* (immunostaining anti-EGFP, green). Scale bars represent 50 μm. In the entire figure, abbreviations are listed in Supplementary Table 4. In low-magnification panels, distance from the most anterior part of the brain is indicated (mm). Images are oriented so that up is dorsal and midline is in the left part of the panels. Red dotted line in **(C)** indicates midline.

To compare adult and perinatal expression of GDNF and GFRα1, we characterized the brain from perinatal *Gfr*α*1*^−(Egfp)/+^; *Gdnf*^−(LacZ)/+^ animals. Representative images of the regions with the expression of both ligand and receptor along the brain were depicted in Supplementary Figure 2 and the hierarchical classification of the structures was described in Supplementary Table 4. Areas with perinatal but without adult GFRα1 expression were imaged using confocal microscopy (Figure 6). To further verify the brain nucleus expressing GDNF, we characterized coronal brain sections from neonatal *Gdnf-Egfp* animals (Supplementary Figure 3).

**Figure 7:**
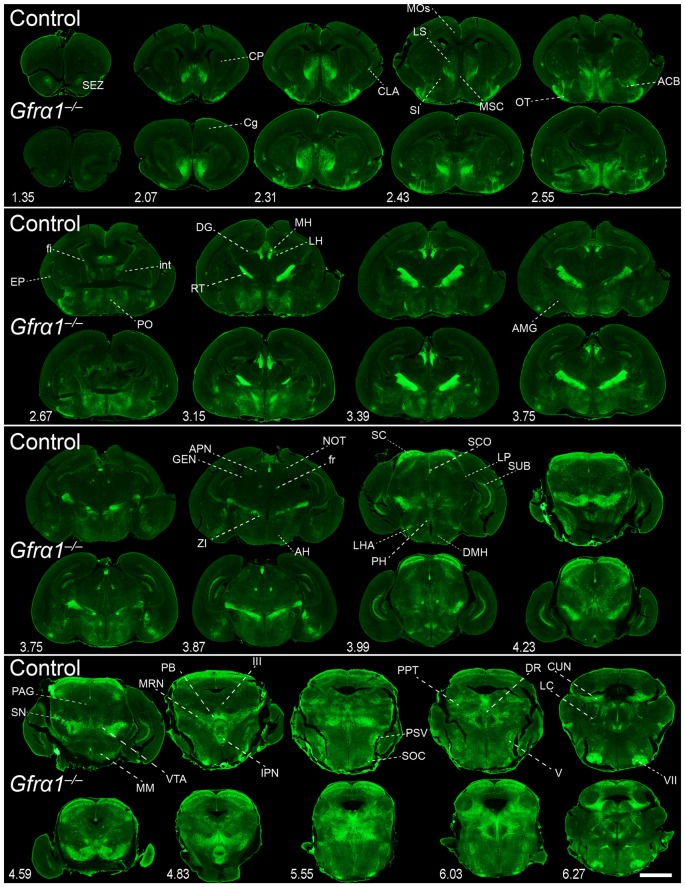
**Absence of brain phenotype in mice lacking GFRα1 at birth.** Coronal brain sections from *Gfrα1^−(Egfp)/+^* (control) and *Gfr*α*1*^−(Egfp)/-(Egfp)^ (*Gfr*α*1*^−/–^; Gfrα1 *knockout*) newborn mice immunostained with an anti-EGFP antibody (green). Scale bar represents 1.2 mm. Abbreviations are listed in Supplementary Table 4 and distance from the most anterior part of the brain is noted (mm).

**Figure 8:**
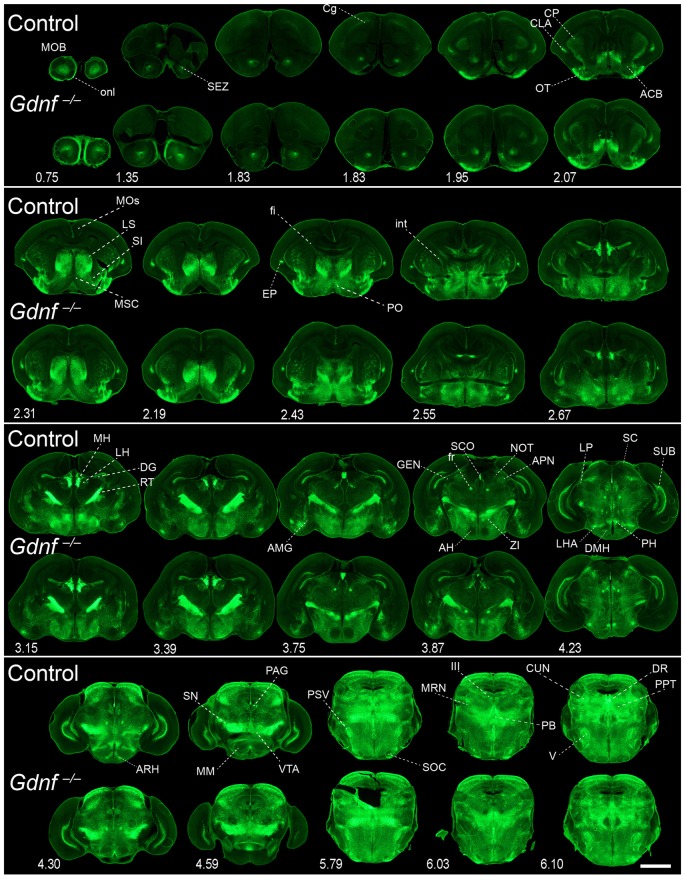
**Absence of brain phenotype in mice lacking GDNF at birth.** Coronal brain sections from *Gfr*α*1*^−(Egfp)/+^; *Gdnf*^−(LacZ)/+^ (control) and *Gfr*α*1*^−(Egfp)/+^; *Gdnf*^−(LacZ)/-(LacZ)^ (*Gdnf* knockout) newborn mice immunostained with an anti-EGFP antibody (green). Scale bar represents 1.2 mm. Abbreviations are listed in Supplementary Table 4 and distance from the most anterior part of the brain is noted (mm).

Our previous work has shown that GDNF expression is very reduced in the brain during embryonic development (Hidalgo-Figueroa et al., 2012). In order to test the relevance of GFRα1 and GDNF function in the maintenance of the cells described in Supplementary Figures 2, 6 and Supplementary Table 4, we took advantage of the reporter activity of the non-functional *Gfr*α*1*^−(Egfp)^ allele, which allowed us to label knocked-out cells. Figure 7 shows cells where GFRα1 has been depleted (embryonic GFRα1 knock-out (KO); *Gfr*α*1*^−(Egfp)/-(Egfp)^) compared with heterozygous (*Gfr*α*1*^−(Egfp)/+^, control) littermates at birth. Similarly, Figure 8 shows the GFRα1 expression pattern in animals lacking GDNF expression (embryonic GDNF KO) that also carry the *Gfr*α*1*^−(Egfp)^ allele, to visualize GFRα1 positive cells (*Gfr*α*1*^−(Egfp)/+^; *Gdnf*^−(LacZ)/-(LacZ)^). Double heterozygous littermates were used as control animals (*Gfr*α*1*^−(Egfp)/+^; *Gdnf*^−(LacZ)/+^). As expected, no major abnormalities in the GFRα1 expression pattern were detected in the absence of trophic support, strongly suggesting that GDNF-GFRα1 signaling pathway is not strongly required during development for the survival of these cells. However, we cannot discard small differences in the number of cells or projections and more quantitative approaches will be required to establish the role of GDNF signaling during development.

### GDNF and GFRα1 Positive Axonal Projections

**Figure 9:**
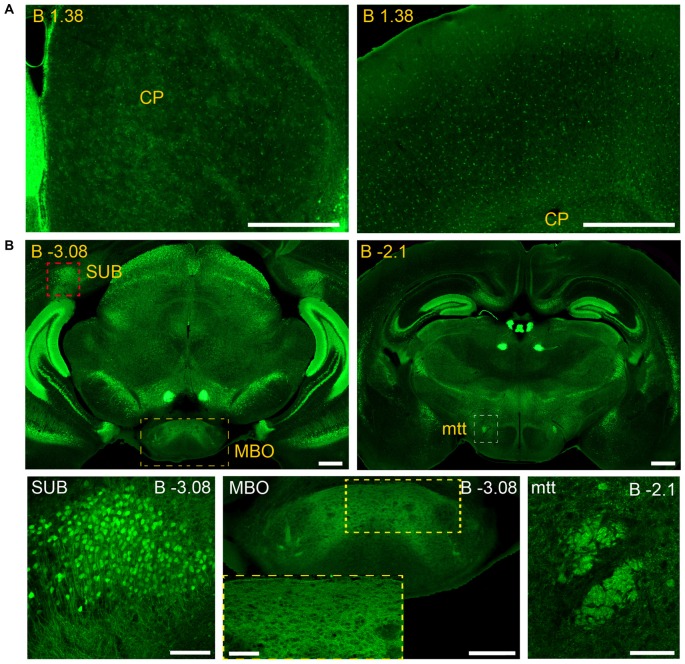
**Long and short-distance trophic GDNF-GFRα1 interactions (I): non-neuronal GFRα1 positive cells; subicular neurons.** Coronal brain sections from adult *Gfr*α*1*^−(Egfp)/+^; *Gdnf*^−(LacZ)/+^ mouse immunostained with an anti-EGFP antibody (green). **(A)** Non-neuronal GFRα1 positive cells homogeneously distributed over the whole brain. CP: Caudoputamen. Scale bars represent 600 μm. **(B)** GFRα1 expression in the mammillothalamic tract (mtt) originated in the SUB and navigating until the mammillary body (MBO). Upper panels (yellow labeled) show low-magnification images. Bottom panels are confocal images (Z-stack projection of 10–20 optical planes of 1 μm thickness) from SUB (red square in upper panel), MBO (yellow square in upper panel) and mtt (red square in upper panel). Yellow square in MBO is shown at high magnification as an inset. Scale bars are 600 μm in upper panels, 200 μm in bottom left, right and center panel-inset, and 400 μm in the center panel. In the entire figure, distance from bregma (B) is indicated (mm).

**Figure 10:**
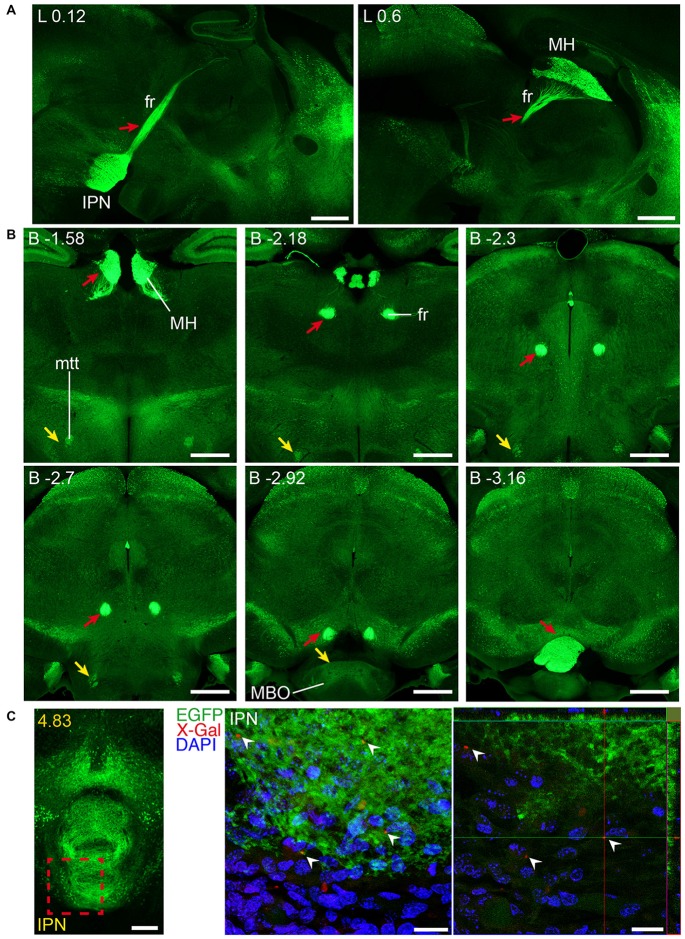
**Long and short-distance trophic GDNF-GFRα1 interactions (II): fasciculus retroflexus.** GFRα1 and GDNF expression in the fasciculus retroflexus tract (fr, red arrows), initiated in medial habenula (MH) neurons projecting to the interpeduncular nucleus (IPN). Sagittal **(A)** and coronal **(B)** brain sections from adult *Gfr*α*1*^−(Egfp)/+^; *Gdnf*^−(LacZ)/+^ mouse immunostained with an anti-EGFP antibody (green). Scale bars are 600 μm. **(C)** Coronal brain section from newborn *Gfrα1^−(Egfp)/+^*; *Gdnf*^−(LacZ)/+^ mouse showing GFRα1 expression (anti-EGFP, green), GDNF expression (X-gal positive, red, white arrowheads) and DAPI staining (blue). Left panel: red square indicates localization of confocal images. Middle panel: confocal Z-stack projection of 10–20 optical planes of 1 μm thickness. Right panel: individual optical plane. Scale bars are 200 μm in left panel and 20 μm in central and right panels. A partial view of the mtt is also visible (yellow arrowheads in **B**). Distance from midline (L) in **(A)**, bregma (B) in **(B)** or the most anterior part of the brain **(C)** is noted (mm).

**Figure 11:**
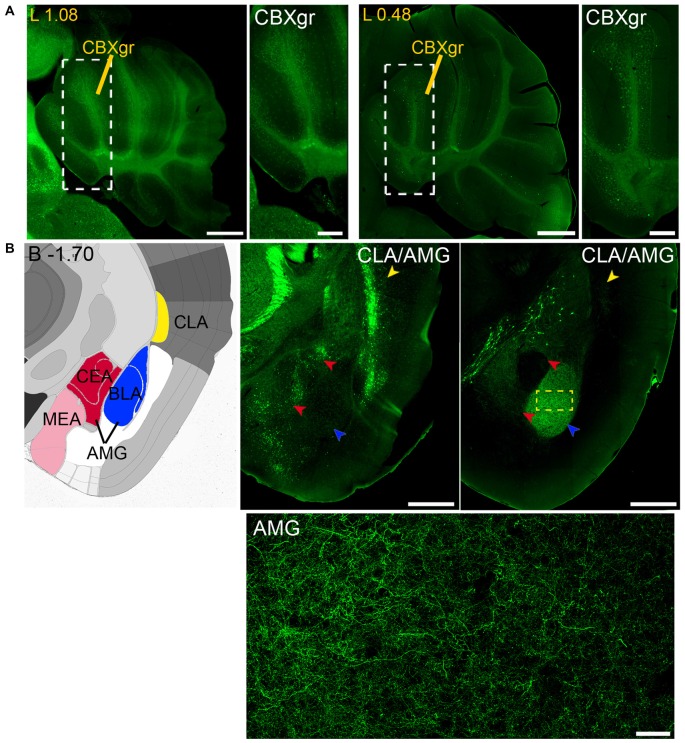
**Long and short-distance trophic GDNF-GFRα1 interactions (III): cerebellum; GDNF positive axons. (A)** GFRα1 and GDNF expression in the granular layer of the cerebellar cortex (CBXgr). Sagittal brain sections from *Gfr*α*1*^−(Egfp)/+^; *Gdnf*^−(LacZ)/+^ (left, 1st and 2nd panels) and *Gdnf-Egfp* (right, 3rd and 4th panels) immunostained with an anti-EGFP antibody (green). Yellow labeled panels: localization of high-magnification images (white square). White labeled panels: high-magnification of CBXgr. Scale bars represent 600 μm in low-magnification images and 200 μm in high-magnification images. Distance from midline (L) is indicated (mm). **(B)** Brain areas with local GFRα1 expression innervated by GDNF positive axons. Left panel: modified scheme from Allen Brain Atlas showing the localization of claustrum (CLA, yellow) and different nuclei of the amygdala (AMG, red, blue and pink). Brain coronal sections from *Gfr*α*1*^−(Egfp)/+^; *Gdnf*^−(LacZ)/+^ (middle panel) and *Gdnf-Egfp* (right panel) immunostained with an anti-EGFP antibody (green). Distance from bregma (B) is indicated (mm). CLA, yellow arrowheads; and AMG, red and blue arrowheads are shown. Bottom panel: confocal Z-stack projection of 10–20 optical planes of 1 μm thickness from the area inside the yellow square in upper-right panel. Scale bars represent 600 μm (upper panels) and 50 μm (bottom panel). Abbreviations are listed in Supplementary Table 3.

Our previous study has identified neurons as the main brain cells expressing GDNF (Hidalgo-Figueroa et al., 2012). Unexpectedly, analysis of *Gfr*α*1*^−(Egfp)/+^; *Gdnf*^−(LacZ)/+^ mice showed that GFRα1 was not only expressed by neurons in the adult brain, but also by non-neuronal cells (Figure 9A). One of the advantages of the technology used here is that, thanks to the uniform distribution of EGFP in neurons, we can describe the brain areas innervated by projection neurons. Previous studies have shown the positive response of substantia nigra compact cells to striatal GDNF (for a review see d’Anglemont de Tassigny et al., 2015), that is produced by local interneurons (Hidalgo-Figueroa et al., 2012) and exerts its action on GFRα1 positive substantia nigra compact axonal terminals. This well-known long-distance trophic interaction was confirmed here (Figures 1–3), validating the approach. In addition, we also observed a GFRα1 positive axonal tract corresponding with the mammilothalamic tract that is initiated in the SUB and reach the mammillary nucleus after passing through GDNF positive areas, including the medial septum and the anterior thalamus (Figure 9B and Supplementary Tables 3, 4). Similarly, GFRα1 positive axons from the medial habenula (MH) reach the interpeduncular nucleus (IPN), GDNF positive during postnatal development (Figure 10), through the fasciculus retroflexus. In the cerebellum, we found another example of local production of GDNF by interneurons in regions containing GFRα1 positive terminals (Figure 11A).

Finally, analysis of *Gdnf-Egfp* mouse brains revealed GDNF positive axons that reach both the claustrum (CLA) and amygdala (AMG; Figure 11B).

### Regions with Simultaneous Local Expression of GDNF and GFRα1

**Figure 12:**
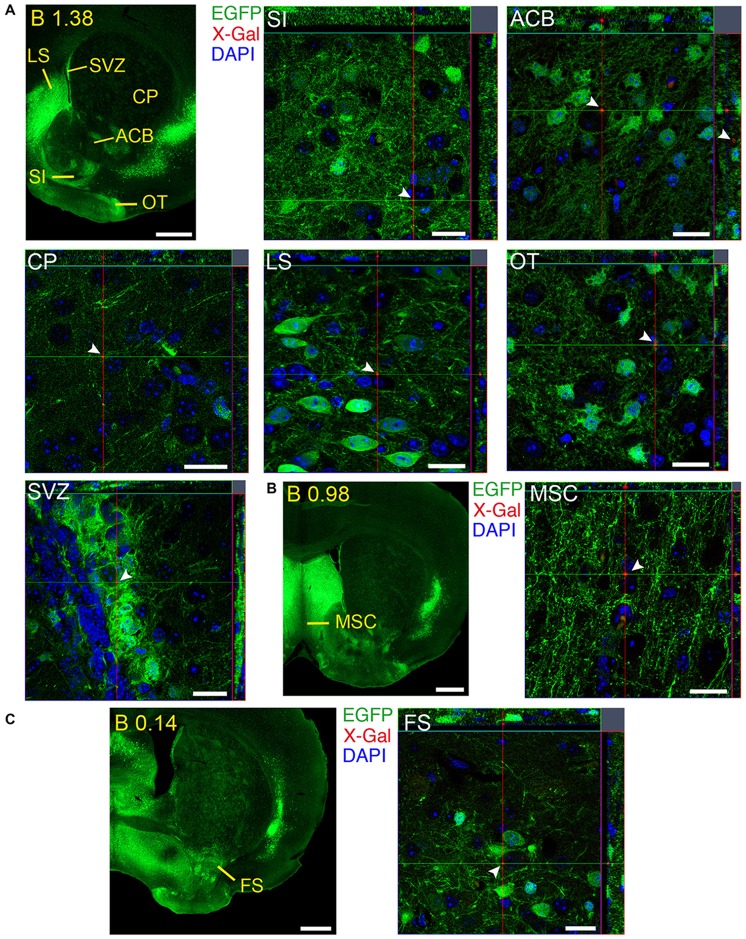
**Brain regions expressing GFRα1 and GDNF in different cell types in the adult brain. (A–C)** Coronal brain sections from adult *Gfr*α*1*^−(Egfp)/+^; *Gdnf*^−(LacZ)/+^ mouse showing GFRα1 (anti-EGFP, green) and GDNF (X-gal positive in red, white arrowheads) expression. Yellow labeled panels indicate localization of areas of interest. White labeled panels are confocal images of the areas of interest from one optical plane. In confocal images, DAPI staining is shown (blue). Scale bars are 600 μm in yellow-labeled panels and 20 μm in confocal images. See Supplementary Table 3 for abbreviations. Distance of the coronal section from bregma (B) is indicated (mm).

**Figure 13:**
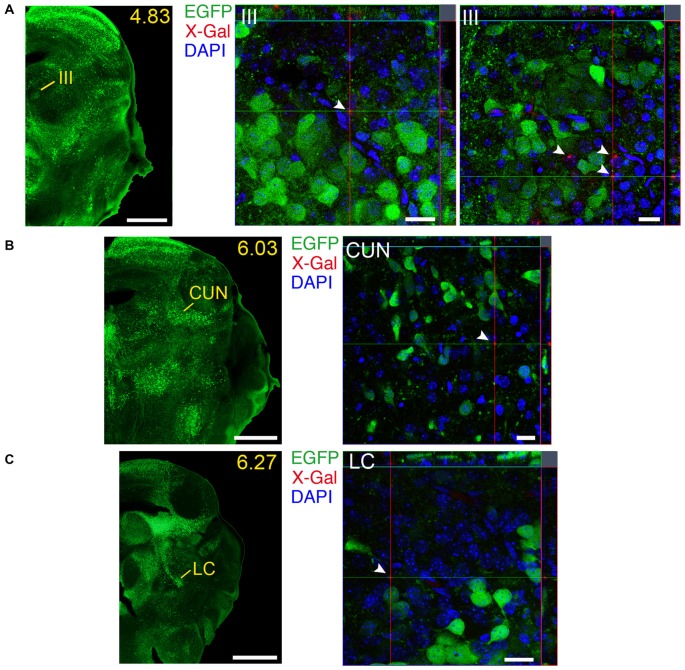
**Brain regions expressing GFRα1 and GDNF in different cell types in the neonatal brain. (A–C)** Coronal brain sections from neonatal *Gfr*α*1*^−(Egfp)/+^; *Gdnf*^−(LacZ)/+^ mouse showing GFRα1 (anti-EGFP, green) and GDNF (X-gal positive, red, white arrowheads) expression. Yellow-labeled panels indicate localization of areas of interest. White labeled panels are confocal images of areas of interest from one optical plane. In confocal images, DAPI staining is shown (blue). Scale bars are 600 μm in yellow-labeled panels and 20 μm in confocal images. CUN, Cuneiform nucleus; III, Oculomotor nucleus; LC, Locus ceruleus. Distance from the most anterior part of the brain (mm) is indicated.

**Figure 14:**
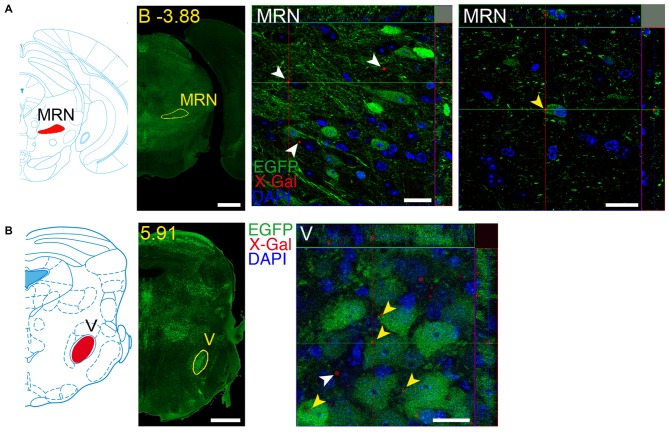
**Brain regions expressing both GFRα1 and GDNF in the same neuron.** Coronal brain sections from adult **(A)** or neonatal **(B)** *Gfr*α*1*^−(Egfp)/+^; *Gdnf*^−(LacZ)/+^ mouse showing GFRα1 (anti-EGFP, green) and GDNF (X-gal positive in, red) expression. Left panels: modified scheme from Franklin and Paxinos (1997) **(A)** or Paxinos et al. (2007) **(B)** showing the localization of areas of interest. Yellow labeled panels: low-magnification coronal brain sections of the same region. White labeled panels are confocal images, one optical plane, of the areas of interest. Yellow arrowheads indicate X-gal deposits inside EGFP positive cell. White arrowheads indicate X-gal deposits inside non-EGFP positive cells. In confocal images, DAPI staining is shown (blue). Scale bars are 600 μM in low magnification and 20 μm in confocal images. MRN, Midbrain reticular nucleus; V, V motor nucleus of trigeminal. Distance of the coronal section is indicated from bregma (B) in panel **(A)**, or from the most anterior part of the brain in panel **(B)** (mm).

We identified several regions with local expression of both GFRα1 and GDNF including brain areas that a previous report has failed to detect (Supplementary Tables 3, 4). Regions with intermingled somatic expression of both the ligand and the receptor include substantia innominata (SI), ACB, caudoputamen, septum, midbrain reticular nucleus (MRN), and olfactory tubercle (OT) in adult animals; and oculomotor nucleus (III), locus ceruleus (LC) and the V motor nucleus of trigeminal in neonatal mice. In order to systematically define the cells expressing both markers, we made a detailed analysis of these areas by confocal microscopy. In most of the adult animals, no single colocalization GDNF-GFRα1 was observed in neurons despite the high level of expression of both markers (Figure 12). Apart from neurons, the secretory cells of the subcomissural organ produced both markers (data not shown). In newborn animals, GFRα1 and GDNF expressing cells were intermingled but no single neuron was found expressing both markers in the III, cuneiform nucleus (CUN) or LC (Figure 13). On the contrary, in the adult MRN and the neonatal V motor nucleus of trigeminal we observed not only neurons expressing both markers but also cells that independently produce either GFRα1 or GDNF (Figure 14).

### GFRα1 and GDNF Expression in The Spinal Cord

**Figure 15:**
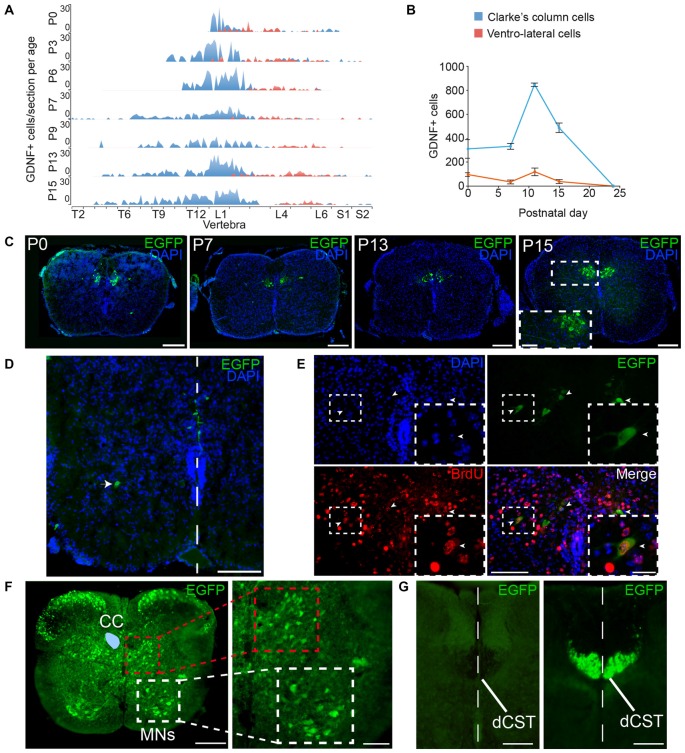
**Expression of GFRα1 and GDNF in the spinal cord (SC). (A,B)** Number of cells expressing GDNF along the SC at several postnatal (P) days as observed in *Gdnf-Egfp* mice immunostained with an against EGFP antibody (green). In blue, Clarke’s column (CC) cells; in red, ventrolateral-located GDNF positive cells. **(A)** number of EGFP positive cells per vertebra. X-axis indicates the vertebrae position in the thoracic (T), lumbar (L) and sacral (S) level. **(B)** Total number of GDNF positive cells. **(C–E)** SC sections from* Gdnf-Egfp* mouse at thoracic level. **(C)** Representative images of CC cells at several postnatal days (anti-EGFP, green, and DAPI, blue). In P15, inset (white square) is shown at high magnification. Scale bars are 200 μm and 80 μm in inset. **(D)** Ventrolateral (white arrowhead) and midline (white line) located populations of GDNF expressing cells (immunostaining anti-EGFP, green, and DAPI, blue) in P0 SC section at lumbar level. Scale bar is 100 μm. **(E)** GDNF positive cells exit cell cycle at 11th embryonic day (E11). Colocalization of BrdU (anti-BrdU, red) and EGFP (anti-EGFP, green) in a P7 descendent from a pregnant female injected with BrdU at E11. Dapi staining is shown in blue. Scale bars are 100 μm and 40 μm in the inset (white squares). Arrowheads indicate BrdU/EGFP double positive cells. **(F)** Expression pattern of GFRα1. SC sections from *Gfr*α*1*^−(Egfp)/+^; *Gdnf*^−(LacZ)/+^ mouse at thoracic level (anti-EGFP, green). CC (blue area drawn over left part) is surrounded by interneurons located in the intermediolateral horn (red square). Motor neurons (MNs) express GFRα1 in ventral horn (white square). Right panel shows high magnification of squared areas. Scale bar is 200 μm in the left panel and 100 μm in the right panel. **(G)** Dorsal corticospinal tract (dCST) is negative for GFRα1 but undergoes recombination in *Emx1-Cre* line. SC sections from *Emx1-Cre/+; Gfrα1^Flox/+^* (left) and *Emx1-Cre/+; R26R-YFP* mice (right) at thoracic level (anti-EGFP, green). The midline is indicated by a white dotted line. Scale bars are 100 μm.

Additionally to the well-documented expression of GFRα1 by the MNs of the SC, GDNF is expressed by Clarke’s column (CC) neurons (Hantman and Jessell, 2010). We characterized the expression pattern of GDNF in newborn *Gdnf-Egfp* mice. According to the anatomical location of CC, GDNF positive cells were found in vertebrae of the thoracic and lumbar levels (Figure 15A). We observed a second type of GDNF positive cells localized in the ventral horn (ventrolateral position) of most caudal vertebrae (mainly lumbar; Figure 15D). These cells could correspond with especial CC cells that have been described in humans in this position (Gray, 1918). Regarding the temporal distribution of the cells, GDNF expression was barely detected in the CC at embryonic day 16 (E16), a peak of maximal expression was observed at postnatal day 12 (P12) and GDNF expression was turned off at P25 (Figures 15A–C and data not shown). At P0, cells with a different morphology were also observed around midline (Figure 15D), which could be reminiscent of the cells described during development to modulate midline crossing of commissural axons (Charoy et al., 2012). In order to define when GDNF positive cells were born, we used BrdU tracing. We injected pregnant *Gdnf-Egfp* mice with BrdU at embryonic days from E9 to E15 and the animals were sacrificed at P7 to analyze GDNF expression. As observed in Figure 15E, the CC cells that will express GDNF during the perinatal period were born at E11.

In order to suggest a possible trophic circuit in the SC, we studied the expression of GFRα1 in the perinatal SC. CC cells send their axon to the cerebellum and receive information from primary sensory neurons, cortical neurons and local GABAergic interneurons (Hantman and Jessell, 2010). Analysis of perinatal *Gfr*α*1*^−(Egfp)/+^ mice indicated that several interneurons localized in the dorsal horn and around the CC are positive for GFRα1 (Figure 15F). As previously described, MNs of the ventral horn also expressed low levels of the receptor (Figure 15F). However, axons coming from the cortex through the dorsal corticospinal tract (dCST), positive for *Emx1* marker, were negative for GFRα1 expression, suggesting that interneurons more than cortical neurons were the main target of the secreted GDNF in the SC (Figures 15F,G). As a control, we verified that genetic labeling with EGFP of cortical cells (Emx1+) projecting to CC could be revealed by our analysis (Figure 15G).

## Discussion

Trophic factors have been postulated as promising therapies for central nervous system (CNS) diseases; however, initial excitement was cooled down by clinical trials, which revealed difficulties in the administration and severe negative effects of the dispensed treatments (Nutt et al., 2003; Tovar-Y-Romo et al., 2014). The translation to the clinics of these therapies will be facilitated by improved knowledge about CNS GDNF-GFRα1 trophic circuits and how brain connectivity is regulated during development by this signaling pathway. In this article, we performed the simultaneous detection of GDNF and GFRα1 expression patterns, complementing previous studies using *in situ* hybridization (Trupp et al., 1997; Golden et al., 1999) or analysis of reporter mice (Pascual et al., 2008; Hidalgo-Figueroa et al., 2012). Our work presents several advantages and caveats. The technique employed here allowed us to simultaneously observe both the soma and projections of the neurons expressing the ligand or the receptor and therefore, to describe the projection neurons that express both molecules. Second, we benefited here from the high sensitivity and stability of the signal from reporter mice. Last, the use of reporter mice allowed us for an exhaustive and detailed analysis that could be crucial for the understanding of the physiology of GDNF and to improve GDNF-based therapies.

A drawback of our method is that transgenic mice could not recapitulate the expression of the endogenous loci, although the use of KI animals decreases the possibility of observing an abnormal pattern of expression. However, our results are highly compatible with analysis published before (Trupp et al., 1997; Golden et al., 1999) and show other expression sites that have been previously missed, probably because their low expression level, the lack of sensitivity of mRNA detection, and the shorter life of mRNA in the cell compared with EGFP stability. The high correlation between these reports and our data validates our results in the analysis of GFRα1 expression pattern, whereas GDNF detection is validated by the analysis and comparison of two different reporter mice and also by the high degree of correlation with *in situ* data (Trupp et al., 1997; Golden et al., 1999). Another drawback in the use of heterozygous KI mice is the presence of only half dose of the gene, a situation that could alter the normal pattern of expression. In our case, we have used double heterozygous mice to map circuits and short-range interactions. However, the expression pattern of both GFRα1 and GDNF is identical in heterozygous individuals than in animals carrying both alleles, strongly arguing against a change in the expression associated with a half dose of both GDNF and GFRα1. A further limitation of our study is that, with the present technique, we can only propose trophic interactions. Studies employing tracers and genetic ablation in specific cells will be required to define the role of the GDNF-GFRα1 signaling pathway in every proposed circuit. Finally, it is noteworthy to remember that although GDNF signals mainly through GFRα1 (Pascual et al., 2011), our study does not address GFRα1 independent signaling.

Adult GFRα1 expression was described in the cerebral cortex, olfactory areas, hippocampal formation, AMG, OT, septum, reticular nucleus of the thalamus, MH, mammillary nucleus, zona incerta, ventral tegmental area, substantia nigra, IPN, dorsal nucleus of the raphe and the cerebellar cortex (Trupp et al., 1997). All these brain nuclei are also reported in our present work, with a greater detail due to the easier identification of the structures using reporter mice. For instance, we defined the specific subareas that contain GFRα1 expressing cells in the cerebral cortex, hippocampal formation, AMG, and septum. New brain areas described here as containing GFRα1 expressing neurons are the CLA, endopiriform nucleus, accumbens, fundus of striatum, SI, anterior division of the bed nuclei of the stria terminalis, lateral posterior nucleus of the thalamus, geniculate complex, anteroperiventricular nucleus, dorsomedial nucleus, periventricular nucleus, superior colliculus, III, MRN, periaqueductal gray, anterior pretectal nucleus, nucleus of the optic tract, pedunculopontine nucleus, principal sensory nucleus of the trigeminal, parabrachial nucleus, superior olivary complex, V motor nucleus of trigeminal, superior nucleus of the raphe and LC. We failed to report expression in the previously described GFRα1 expressing nuclei: the glomerular layer of the olfactory areas, red nucleus, Purkinje and molecular layer of the cerebellum and deep cerebellar nuclei (Trupp et al., 1997). The differences between both studies can be due to the different species analyzed, rat in Trupp et al. (1997) and mouse in the present work. Interestingly, our data are in almost perfect agreement with those of the Allen Brain Atlas. Exceptions included the prefrontal cortex, where *in situ* hybridization detected expression that we did not reproduce and the periventricular nucleus, where we observed GFRα1 expression that was not confirmed by the data found in the Allen Brain Atlas.

Regarding adult GDNF expression, a good match was observed between previous works and our data (Trupp et al., 1997; Pascual et al., 2008; Hidalgo-Figueroa et al., 2012). New GDNF expressing areas described here are the fundus of striatum, SI, nucleus of reunions, rhomboid nucleus, geniculate complex, parasubthalamic nucleus, superior colliculus, periaqueductal gray and pedunculopontine nucleus. In this case, *in situ* from Allen Brain Atlas did not reproduce the previously published data (Trupp et al., 1997; Pascual et al., 2008; Hidalgo-Figueroa et al., 2012) and therefore was not considered in this work. Similarly, a good agreement was found between previously described neonatal GDNF and GFRα1 expression patterns (Golden et al., 1999; Hidalgo-Figueroa et al., 2012) and our present work, although a greater detail into the substructures is provided here (Supplementary Table 4).

Apart from the systematic description of GDNF and GFRα1 expressing areas, our study also reveals that in both perinatal and mature brains, GFRα1 showed wider expression than GDNF. Although this was reported (Trupp et al., 1997), it became more evident in our work due to the visualization of the GFRα1 axonal fibers (EGFP positive). In particular, the hippocampal formation, midbrain, and hindbrain are regions containing a high number of GFRα1 positive neurons but very localized or even undetectable (hippocampus) expression of GDNF. The low GDNF brain levels (d’Anglemont de Tassigny et al., 2015) and the precise localization of GDNF expressing cells suggest a tight control in their expression. Striatal GDNF levels are repressed by Sonic hedgehog (Gonzalez-Reyes et al., 2012) and several adverse effects have been associated with GDNF treatment (d’Anglemont de Tassigny et al., 2015), highlighting the importance of a tight control of brain GDNF levels.

GDNF expression pattern is broader in adult than in perinatal animals (see below). However, GFRα1 pattern is wider in perinatal than in adults. Whether this apparent contradiction is a developmental requirement or a consequence of how the developmental program refines the expression patterns is not known. Genetic deficiency of either the ligand or the receptor did not produce obvious abnormalities, indicating a role for GDNF signaling in adult more than during embryogenesis. That is also in agreement with previous reports describing low GDNF expression in brain during development (Golden et al., 1999; Hidalgo-Figueroa et al., 2012) and the minimal neurological phenotype observed in mice lacking either GDNF (Moore et al., 1996) or GFRα1 (Cacalano et al., 1998). However, several roles for GDNF have been described during this period, including an inductive role for dopaminergic neurons of the substantia nigra compact part (Peng et al., 2011) and the maintenance of some sensory and MNs (Moore et al., 1996; Cacalano et al., 1998), suggesting that more detailed studies will be required to identify subtle defects.

GDNF was isolated from an immortalized glial cell line (Lin et al., 1993), however, previous works revealed that GDNF expression is mainly neuronal in the CNS (Hidalgo-Figueroa et al., 2012). On the contrary, non-neuronal cells expressed GFRα1, strongly suggesting that GDNF could exert a trophic action on this cell population. In a fair agreement, GDNF protects astrocytes against ischemia (Yu et al., 2007) through ERK/NFκB signaling pathway (Chu et al., 2008) and the role of non-neuronal cells in the protection achieved by GDNF in several experimental models should be now be taken into account.

Our studies suggested two types of trophic relations, involving either long- or short-distance interactions between ligand and receptor expressing cells. Long-distance trophic support has been described in both the nigrostriatal and mesolimbic trophic circuits (Pascual et al., 2011), involving retrograde transport of GDNF (Tomac et al., 1995; Ibáñez, 2007). Expression of GFRα1 in the MH has been previously notified (Trupp et al., 1997; Glazner et al., 1998; Golden et al., 1999), although the source of the trophic factor has not been identified so far. We described here that GFRα1 positive projecting neurons from the MH send their axons through the fasciculus retroflexus to innervate the IPN, whereas no expression was observed in the axons that innervate the raphe complex, another prominent efferent pathway of the MH (Herkenham and Nauta, 1979). The IPN expresses GDNF during perinatal life and could account for specification or trophic support of habenular neurons during this developmental period. The fact that GFRα1 expression in this tract is also observed in adult animals suggests a putative role of trophic signaling in pathology. In addition to the MH, GFRα1 positive projection neurons from the SUB send their axons through the fimbria, fornix, and postcommissural fornix, to reach the mammillary bodies through the mammillary tract (Raisman et al., 1966; Poletti and Creswell, 1977). This axonal tract splits to reach anterior thalamus where we observe GFRα1 positive axons, however, with the present technique, we cannot determine whether thalamic terminals belong to subicular neurons and further analysis using tracers will be required. On the contrary to the MH/IPN, the mammillary body (MBO) do not express GDNF.

So far local production of GDNF by interneurons has been described to signal through GFRα1 positive axonal terminals of distantly located neurons (Hidalgo-Figueroa et al., 2012). However, we found brain regions where GDNF could be anterograde transported to the target neurons, a process that was previously proposed for several trophic factors (von Bartheld et al., 2001). A bundle of GDNF positive axons descends through the internal capsule to innervate the AMG and the CLA, where we found GFRα1 expressing interneurons. GDNF expression is scarce in the brain and, therefore, is tempting to speculate that the axons that profusely innervate the basolateral AMG and barely the CLA were originated at the rhomboid nucleus of the thalamus (RH). RH efferent axons innervate both regions (Vertes et al., 2006, 2015) and also send projections to the OT, lateral septum, ACB, and caudate-putamen, all of them containing GFRα1 positive neurons or axons. As an alternative hypothesis, local GFRα1 axons are also observed at the level of both the RH and nucleus of reunions and, therefore, local trophic support could be anticipated.

Short-distance trophic interactions were observed in several adult brain regions. Areas with intermingled expression of GDNF positive neurons and GFRα1 positive cells include ACB, lateral septum, OT, and SI. It is noteworthy that we did not observe cellular colocalization of both the ligand and the receptor, apart from the adult MRN.

Some of the long- and short-distance adult trophic connections described here participate in the limbic circuits mediating addiction (Lüthi and Lüscher, 2014), including the fasciculus retroflexus (Velasquez et al., 2014), the projections from RH to innervate the basolateral AMG, CLA, tACB, ventral tegmental area, OT and lateral septum (Lüthi and Lüscher, 2014). Therefore, a conclusion from our work is that GDNF and GFRα1 are highly expressed in limbic structures, showing an unexpected complexity in both the number of cells and the circuits involving trophic factor expression. Our results suggest that the involvement of GDNF in the regulation of addiction-related circuits is far more sophisticated than just the maintenance of the mesolimbic pathway, as previously proposed (Airavaara et al., 2004; He et al., 2005; Pascual et al., 2008, 2011).

In addition to basal ganglia and limbic systems, GDNF has been described as important for MNs (Bohn, 2004). In the neonatal brain, several cranial MNs populations have been shown to express GFRα1, including III and trigeminal (V) nucleus (Mikaels et al., 2000). Mice lacking either GFRα1 or GDNF present a similar decrease in the number of trigeminal MNs (Moore et al., 1996; Cacalano et al., 1998), however, no effect has been observed in oculomotor neurons in mice deficient for GDNF (Oppenheim et al., 2000). Although it is generally believed that GDNF is transported from the muscle to MNs, approaches using KI animals to follow GDNF expression in muscle have failed to detect it (Whitehead et al., 2005). Both motor nuclei contain GDNF expressing cells (Hidalgo-Figueroa et al., 2012) and our results indicate a particular situation in the V motor nucleus, where expression of GDNF and GFRα1 co-exist in the same neurons. To our knowledge, this is the first report of a possible autocrine mechanism to maintain a neuronal population in the CNS. A similar autocrine trophic mechanism has been proposed for the glomus cells of the carotid body (Villadiego et al., 2005; Pascual et al., 2008), a small organ placed at the bifurcation of the carotid artery responsible for blood oxygen sensing (Weir et al., 2005).

Another territory where GDNF was proposed as a therapeutic agent is the SC. Original reports describing the trophic support of MNs by GDNF (Oppenheim et al., 1995, 2000) were followed by pre-clinical studies in animal models and clinical trials (Tovar-Y-Romo et al., 2014). GDNF expression in the perinatal SC has been described before (Nosrat et al., 1996; Hantman and Jessell, 2010), but the pattern of expression of GFRα1 has not been previously addressed. Additionally to MNs, interneurons surrounding CC are GFRα1 positive and these interneurons have been proposed as involved in the corticospinal corollary circuit regulating motor planning and evaluation (Hantman and Jessell, 2010).

GDNF-based therapies designed to improve several neuronal disorders should take into consideration our findings to improve the ratio between positive patient’s outcome and adverse effects. Further work will be required to unveil the logic of trophic maintenance by the identification of the cell types expressing both GDNF and GFRα1 in each specific brain area. That will also allow for genetic manipulation of both proteins to study their role in adult CNS function.

## Author Contributions

Both authors approved the final version of this article. Experiments were conceived and designed by CO-dSL and AP. CO-dSL performed almost all the experiments. AP performed confocal imaging of EGFP. CO-dSL and AP performed the data analysis and contributed to the manuscript and figure preparation.

## Funding

The research was funded by the Spanish Ministry of Science and Education (SAF2012-33816 and SAF2015-64111-R) and by the grant CTS-2138 from the Regional Ministry of Economía, Innovación, Ciencia y Empleo, co-funded by CEC and FEDER funds. Support from the Spanish Ministry of Education for CO-dSL (predoctoral fellowship Programa FPU, AP2010-1598) is also acknowledged.

## Conflict of Interest Statement

The authors declare that the research was conducted in the absence of any commercial or financial relationships that could be construed as a potential conflict of interest.
