# Supplementary material for: Simultaneous Detection of Both GDNF and GFRα1 Expression Patterns in the Mouse Central Nervous System
Source: Front Neuroanat. 2016 Jun 24;10:73. doi: 10.3389/fnana.2016.00073 (PMC4919337; doi:10.3389/fnana.2016.00073)
Supplement: Supplementary file 1 [file Presentation_1.pdf]

Supplementary Material

Simultaneous Detection Of Both GDNF And GFRα1 Expression Patterns In The Mouse Central Nervous System

Clara Ortega-de San Luis[1] and Alberto Pascual[1,]*

*** Correspondence:** Alberto Pascual; apascual-ibis@us.es

1 Supplementary Figures and Tables

1.1 Supplementary Figures

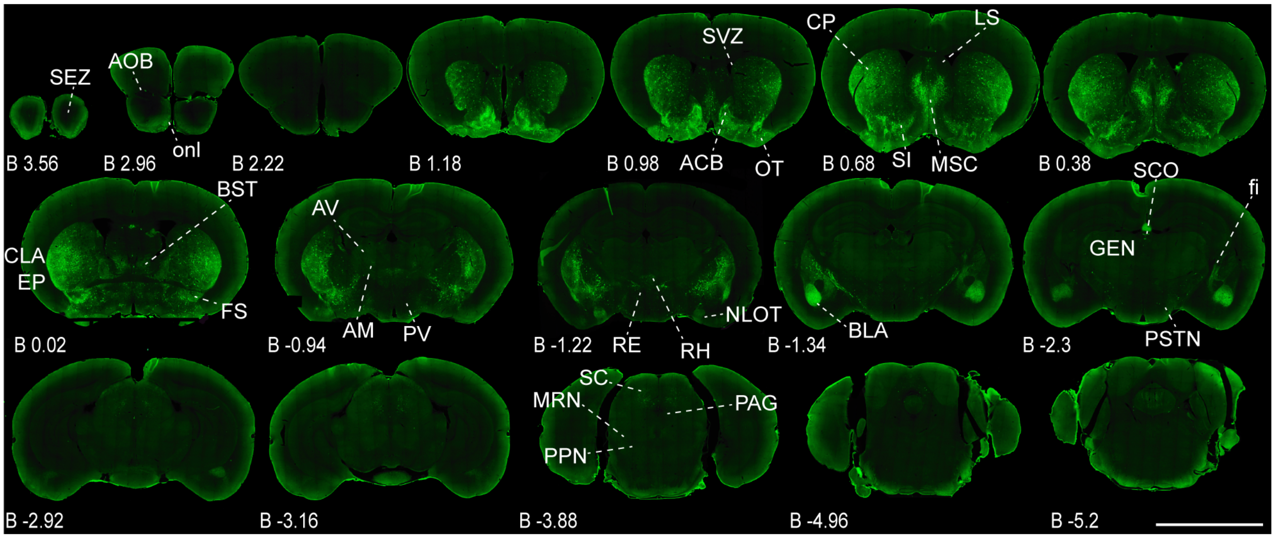

Supplementary Figure 1. GDNF expressing areas in *Gdnf-Egfp* adult mouse. Immunostaining anti-EGFP (green) in coronal brain sections (50 µm thickness) from 1 month old *Gdnf-Egfp* mouse. Scale bar represents 5 mm. Distance from bregma (B) is indicated (mm). Abbreviations are listed in Supp. Table 3.

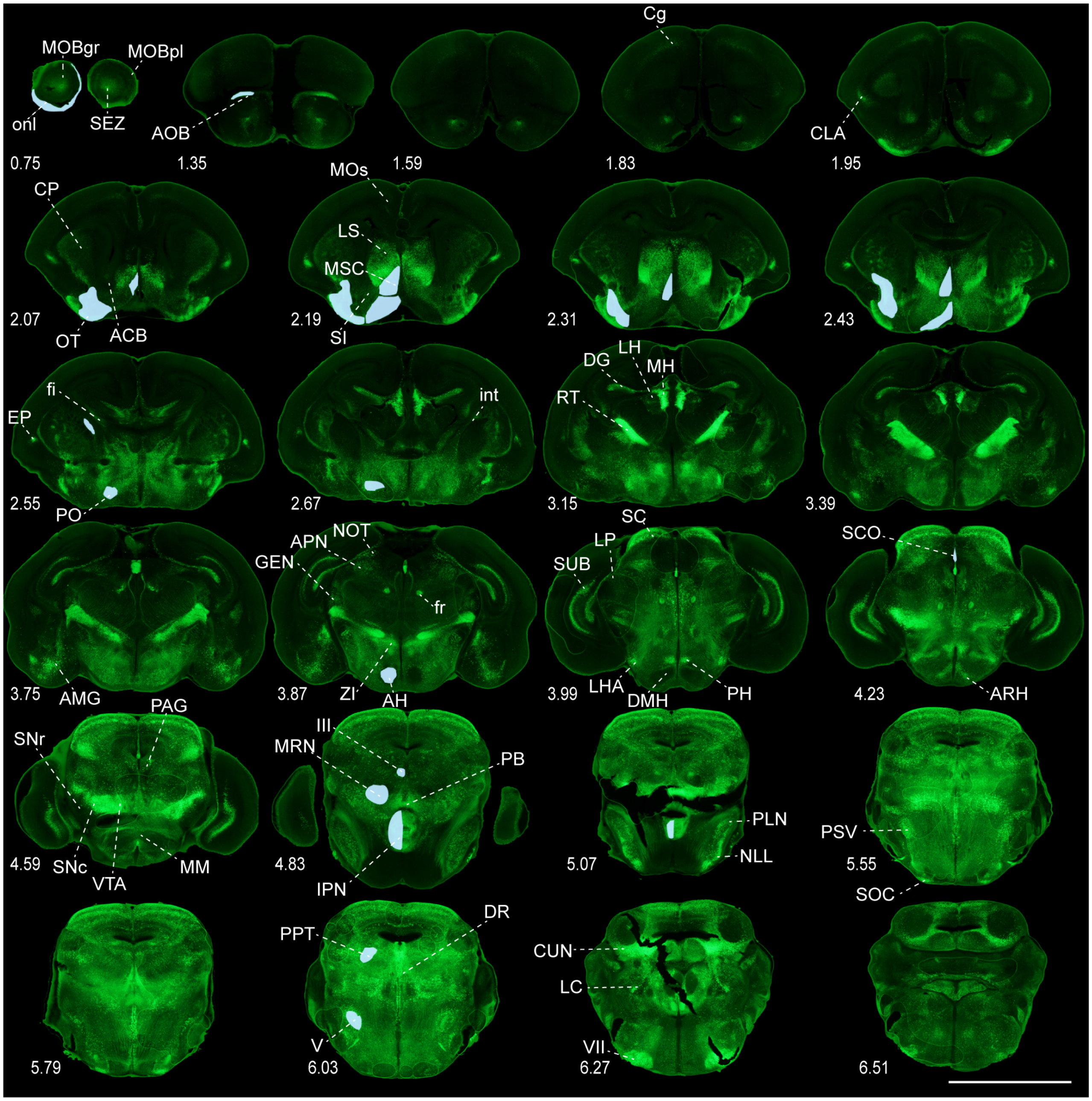

Supplementary Figure 2. Perinatal expression of GFRα1 and GDNF. Coronal brain sections from *Gfrα1* $^{-(Egfp)/+}$; *Gdnf* $^{-(LacZ)/+}$ mice brain showing GFRα1 (anti-EGFP, green) and GDNF expression (X-gal positive, blue areas drawn over left hemispheres). Scale bar represents 2.5 mm. Abbreviations are listed in Supp. Table 4. Distance from the most anterior part of the brain (mm) is shown.

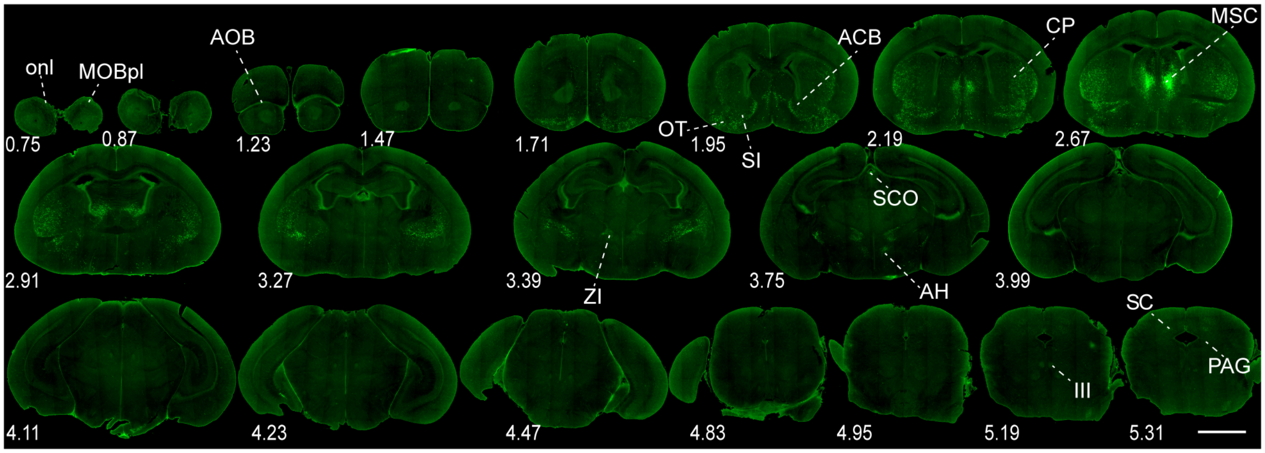

Supplementary Figure 3. GDNF expressing areas in *Gdnf-Egfp* perinatal mouse. Immunostaining anti-EGFP (green) in coronal brain sections (50 µm thickness) from P0 *Gdnf-Egfp* mouse. Scale bar represents 1.2 mm. Distance from the most anterior part of the brain is indicated (mm). Abbreviations are listed in Supp. Table 4.

1.2 Supplementary Tables

Supplementary Table 1. Mouse Lines Used in This Study

Mouse line	Mutations	Objective	Figure correspondence
Gdnf $^{-(LacZ)/+}$; *Gfrα1* $^{-(Egfp)/+}$	GDNF+ cells express LacZ marker; GFRα1+ cells express EGFP marker.	Description of expression patterns.	1A (left and center), 1B, 2, 3, 4A (center), 4C (even panels), 4D (2nd, 4th and 5th panels), 4E (2nd panel), 5A (2nd, 3rd, 4th and 7th panels), 5C (5th panel), 5D (2nd panel), 6A-E, 6F (middle panel), 9, 10, 11A (left), 11B (middle panel), 12, 13, 14, 15F and S2.
Gdnf-Egfp	GDNF+ cells express EGFP marker.	Description of expression pattern.	1A (right), 4A (3rd panel), 4B (2nd panel), 4C (odd panels), 4D (3rd, 6th panels), 4E (3rd and 4th panels), 5A (5th, 6th and 8th panels), 5B (2nd panel), 5C (2nd, 3rd, 4th and 6th panels), 5D (3rd and 4th panels), 6F (3rd panel), 11A (right), 11B (upper-right panel, bottom panel), 15A-E, S1 and S3.
Th-IRES-Cre; Gfrα1 $^{Flox/+}$	Germline cells express CRE recombinase	Generation of *Gfrα1* $^{-(Egfp)}$ allele.	1A (left).
Gfrα1 $^{-(Egfp)/-(Egfp)}$	*Gfrα1* deficient mouse; GFRα1+ cells express EGFP marker.	Analysis of GFRα1 function during development.	7.
Gdnf $^{-(LacZ)/-(LacZ)}$; *Gfrα1* $^{-(Egfp)/+}$	*Gdnf* deficient mouse; GFRα1+ cells express EGFP marker.	Analysis of GDNF function during development.	8.
Emx1 $^{Cre/+}$; *Gfrα1* $^{Flox/+}$	EMX1+ cells recombine *Gfrα1* and express EGFP marker.	Analysis of GFRα1+ cells in dorsal corticospinal tract.	1A (left), 15G (left panel).
Emx1 $^{Cre/+}$; *R26R-YFP*	EMX1+ cells express YFP marker.	Control of recombination in EMX1+ cells.	15G (right panel).

Supplementary Table 2. Primary Antibody Used in This Study

Antibody	Immunizing antigen	Host species	Manufacturer / catalog No.	RRID#	Dilution
Green fluorescent protein (GFP)	Recombinant GFP protein	Rabbit polyclonal	Invitrogen A11122	AB_2307355	1:1000
Bromodeoxyuridine (BrdU)	Non described	Rat monoclonal [BU1/75 (ICR1)]	Abcam; ab6326	AB_305426	1:200
Neuronal Nuclei (NeuN)	Purified cell nuclei from mouse brain	Mouse monoclonal (Clone A60)	Millipore MAB377	AB_11210778	1:500

Supplementary Table 3. **GFRα1 and GDNF expression in adult (P30) mouse brain**

The study is based on at least three brains analyzed per structure. [1] Semi-quantitative analysis of the expression of GFRα1 and GDNF. –: None GFRα1/GDNF positive cell found; +: 1-20 GFRα1 positive / 1-5 GDNF positive; ++: 20-50 GFRα1 positive / 5-20 GDNF positive; and +++: more than 50 GFRα1 positive / more than 20 GDNF positive cells/analyzed structure. Cells are classified as Neurons when expression of NeuN was detected. [2] Semi-quantitative analysis of the expression of GFRα1 and GDNF in projections identified by morphology. High levels, ++; low, +; and absence of signal, –. [3] Discrepancies between LacZ and EGFP reporter mice are shown separated by a slash. [4] References where expression of GFRα1 and GDNF has been reported, 1: Trupp et al. 1997; 2: Hidalgo-Figueroa et al. 2012; 3: Pascual et al. 2008. GFRα1 and GDNF references are separated by a slash. [5] Concordance with Allen Brain Atlas GFRα1 *in situ* hybridization data. +: detected area; –: non detected area. n.a. not applicable.

**P30**								
**Structure**	**Abbrevia-tion**	**Figures**	**GFRα1**		**GDNF (X-gal/EGFP[3])**		**Referen-ces [4]**	**Allen Brain Atlas [5]**
			**Cells [1]**	**Projections[2]**	**Cells [1]**	**Projections [2]**	**(GFRα1 / GDNF)**	**(GFRα1)**
**Cerebral cortex**							1/n.a.	
Somatosensory areas	SS	1B	Neurons (+)	–	–	–	/n.a.	+
Secondary motor area	MO	1B	Neurons (+)	–	–	–	/n.a.	+
Auditory area	AUD	1B	Neurons (+)	–	–	–	/n.a.	+
Visual areas	VIS	1B	Neurons (+)	–	–	–	/n.a.	+
Retrospenial area	RSP	1B, 2I	Neurons (+)	–	–	–	/n.a.	+
Posterior parietal association areas	PTLp	1B	Neurons (+)	–	–	–	/n.a.	+
**Olfactory areas**							1/2	
Main olfactory bulb, outer plexiform layer	MOBopl	1B, 2A	Cells (+)	–	–	–	1/n.a.	+
Accessory olfactory bulb	AOB	1B, 2A, 4B	–	++	Cells (–/+)	–	n.a./	n.a.
Nucleus of the lateral olfactory tract	NLOT	1B, 5A, S1	–	–	–	n.a./+	n.a./n.a.	n.a.
**Hippocampal formation**							1/n.a.	
Field CA1 / CA2	CA1/CA2	1B, 2G	Neurons (+)	–	–	–	/n.a.	+
Field CA3	CA3	1B, 2G						
– Stratum oriens			–	+	–	–	n.a./n.a.	–
– Pyramidal layer			Cells (++)	–	–	–	/n.a.	+
Dentate gyrus	DG	1B, 2I	Neurons (++)	–	–	–	/n.a.	+
Entorhinal area, layer V	ENT	1B, 3A	Neurons (+)	–	–	–	/n.a.	+
Subiculum	SUB	1B, 3C, D, 9B	Neurons (++)	–	–	–	/n.a.	+
**Cortical subplate**								
Claustrum	CLA	1B, 2C, 11B, S1	Neurons (++)	–	–	n.a./+	/n.a.	+
Endopiriform nucleus	EP	1B, 2C, S1,	Neurons (++)	–	–	n.a./+	/n.a.	+
**Amygdala**							1/n.a.	
Central amygdalar nucleus	CEA	1B, 11B	Neurons (+)	–	–	–	/n.a.	+
Medial amygdalar nucleus	MEA	1B, 3A, 11B	Neurons (+)	–	–	–	/n.a.	+
Basolateral amygdalar nucleus	BLA	1B, 11B, S1	–	–	–	n.a./++	n.a./n.a.	n.a.
**Striatum**								
Caudoputamen	CP	1B, 4C, 9A, 12A, S1	–	++	Neurons (+++)	–	n.a./1, 2, 3	n.a.
Accumbens	ACB	1B, 2D, 4C, 12A, S1	Neurons (++)	++	Neurons (++)	–	/1, 2	+
Fundus of Striatum	FS	1B, 2E, 4E, 12C, S1	Neurons (++)	–	Neurons (++)	–		+
Olfactory tubercle	OT	1B, 2C, 2D, 4A, 12A, S1	Neurons (++)	–	Neurons (+++)	–	1/1	+
Lateral septal nucleus	LS	1B, 2D, 2E, 4D, 12A, S1	Neurons (+++)	–	Neurons (+)	–	1/2, 3	+
**Pallidum**							1/2	
Substantia innominata	SI	1B, 2D, 4A, 12A, S1	Neurons (++)	–	Neurons (++)	–		+

Medial septal complex	MSC	1B, 4D, 12B, S1	–	+	Neurons (+++)	–	1/2	n.a.
Bed nuclei of the stria terminalis; anterior division	BST	1B, 2E, 4E, S1	Neurons (++)	–	–	n.a./+	/n.a.	+
**Thalamus**				++			/2	
Lateral posterior nucleus thalamus	LP	1B, 3A	Neurons (++)	–	–	–	/n.a.	+
Anteroventral nucleus thalamus	AV	1B, 5A, S1	–	–	Neurons (+++/+)	–	n.a./1, 3	n.a.
Anteromedial nucleus thalamus	AM	1B, 5A, S1	–	–	Neurons (+++/+)	–	n.a./1, 3	n.a.
Nucleus of reunions	RE	1B, 5A, S1	–	–	Neurons (++)	–	n.a./	n.a.
Rhomboid nucleus	RH	1B,5A, S1	–	–	Neurons (++)	–	n.a./	n.a.
Reticular nucleus thalamus	RT	1B, 2G	Neurons (+++)	–	–	–	1/n.a.	+
Geniculate complex	GEN	1B, 3A, 5C, S1	Neurons (+)	–	Neurons (–/+)	–		+
Medial habenula	MH	1B, 2G, 10	Neurons (+++)	–	–	–	1/n.a.	+
**Hypothalamus**							1/2	
Anteroventral periventricular nucleus	AVPV	1B, 2E	Neurons (++)	–	–	–	/n.a.	+
Dorsomedial nucleus	DMH	1B, 2G	Neurons (++)	–	–	–	/n.a.	+
Periventricular nucleus	PV	1B, 3A, 5C	Neurons (++)	–	–	n.a./+	/n.a.	–
Parasubthalamic nucleus	PSTN	1S, 5C	–	–	Neurons (–/+)	–	n.a./	n.a.
Mammillary nucleus	MBO	1B, 9B, 10B	–	++	–	–	n.a./n.a.	+
Posterior hypothalamic nucleus	PH	1B, 3A	Neurons (++)	–	–	–	/n.a.	+
Tuberal nucleus	TU	1B, 2H	Neurons (+)	–	–	–	/n.a.	+
Zona incerta	ZI	1B, 2F	Neurons (++)	–	–	–	1/n.a.	+
**Midbrain**			–	++				
Superior colliculus	SC	1B, 3B, 5D, S1	Neurons (++)	++	Neurons (–/+)	–		+
Oculomotor nucleus	III	1B, 3E	Neurons (++)	–	–	–	/n.a.	+
Midbrain reticular nucleus	MRN	1B, 3D, 14A	Neurons (++)	–	Neurons (++)	–	/2	+
Ventral tegmental area	VTA	1B, 3C	Neurons (++)	–	–	–	1/n.a.	+
Periaqueductal gray	PAG	1B, 3D, 5D, S1	Neurons (+)	+	Neurons (+/++)	–		+
Substantia nigra							1/2	
– Compact part	SNc	1B, 3C	Neurons (++)	–	–	–	1/n.a.	+
– Reticular part	SNr	1B, 3C	Neurons (++)	–	–	–	1/n.a.	+
Anterior pretectal nucleus	APN	1B, 3A, C	Neurons (+)	–	–	–	/n.a.	+
Nucleus of the Optic tract	NOT	1B, 3C	Neurons (+)	–	–	–	/n.a.	+
Pedunculopontine nucleus	PPN	1B, 3F, AC, S1	Neurons (+)	+	Neurons (+)	–		+
Interpeduncular nucleus	IPN	1B, 10	–	+++	–	–	1/n.a.	n.a.
Dorsal nucleus raphe	DR	1B, 3F	Neurons (++)	–	–	–	1/n.a.	+
**Hindbrain**								
Principal sensory nucleus of the trigeminal	PSV	1B, 3F	Neurons (++)	–	–	–	/n.a.	+
Parabrachial nucleus	PB	1B, 3G	Neurons (+)	–	–	–	/n.a.	+
Superior olivary complex	SOC	1B, 3G	Neurons (++)	–	–	–	/n.a.	+
V Motor nucleus of trigeminal	V	1B, 3H	Neurons (++)	–	–	–	/n.a.	+
Superior central nucleus raphe	CS	1B, 3F	Neurons (++)	–	–	–	/n.a.	+
Locus ceruleus	LC		Neurons (++)	–	Neurons (+)	–	/1, 2	+
**Cerebellum**								
Cerebellar cortex, granular layer	CBXgr	11A	–	++	Neurons (–/+)	–	n.a./1	+
**Fiber tracts**								
Olfactory nerve layer of main olfactory bulb	onl	1B, 2A, 4A, S1	–	++	–	n.a./+	n.a./n.a.	n.a.
Optic tract	opt	1B	–	+	–	–	n.a./n.a.	n.a.
Corpus callosum, anterior forceps	fa	1B, 2C	–	+	–	–	n.a./n.a.	n.a.
Internal capsule	int	1B	–	+	–	–	n.a./n.a.	n.a.
Fimbria	fi	1B, 2G, 5B, S1	–	++	–	n.a./+	n.a./n.a.	n.a.
Columns of the fornix	fx	1B, 2E	–	++	–	–	n.a./n.a.	n.a.
Stria terminalis	st	1B	–	+	–	–	n.a./n.a.	n.a.
Mammilothalamic tract	mtt	1B, 2G, 9B, 10B	–	+	–	–	n.a./n.a.	n.a.
Fasciculus retroflexus	fr	1B, 10	–	+++	–	–	n.a./n.a.	n.a.

**Others**								
Subependymal zone	SEZ	1B, 2A, 4A, S1	Cells (++)	–	Cells (+)	–		+
Subventricular zone	SVZ	1B, 2D, 4D, 12A, S1	Cells (++)	–	Cells (+)	–		+
Subcommisural organ	SCO	1B, 3A, 5C, S1	Cells (+++)	–	Cells (+++)	–	/1, 2	+

Supplementary Table 4. GFRα1 and GDNF expression in neonatal (P0) mouse brain

The study is based on at least three brains analyzed per structure. [1] Semi-quantitative analysis of the expression of GFRα1 and GDNF –: None GFRα1/GDNF positive cell found; +: 1-20 GFRα1 positive / 1-5 GDNF positive; ++: 20-50 GFRα1 positive / 5-20 GDNF positive; and +++: more than 50 GFRα1 positive / more than 20 GDNF positive cells/analyzed structure. [2] Semi-quantitative analysis of the expression of GFRα1 and GDNF in projections identified by morphology. High levels, ++; low, +; and absence of signal, –. [3] Discrepancies between LacZ and EGFP reporter mice are shown separated by a slash. [4] References where expression of GFRα1 and GDNF has been reported. 1: Golden et al. 1999; 2: Hidalgo-Figueroa et al. 2012. [5] Concordance with Allen Brain Atlas GFRα1 *in situ* hybridization data. +: detected area; –: non detected area. n.a. not applicable. n.i.: structure not identified in the Allen Brain Atlas.

**P0**								
**Structure**	**Abbre-viation**	**Figures**	**GFRα1**		**GDNF (X-gal/EGFP[3])**		**References [4]**	**Allen Brain Atlas [5]**
			**Cells[1]**	**Projections[2]**	**Cells[1]**	**Projections[2]**	**(GFRα1 / GDNF)**	**(GFRα1)**
**Cerebral cortex**							1/n.a.	
Cingulate cortex	Cg	S2	++	–	–	–	/n.a.	+
Motor cortex, secondary	MOs	S2	++	–	–	–	/n.a.	n.i.
**Olfactory areas**							1/1, 2	
Main olfactory bulb, granular cell layer	MOBgr	S2	+	–	–	–	/n.a.	+
Outer plexiform layer	MOBpl	S2, S3	+	–	–/+	–		+
Accessory olfactory bulb	AOB	S2, S3	–	++	–	n.a./+	n.a./n.a.	–
**Hippocampal region**		S2					1/ n.a.	
Dentate Gyrus	DG	S2	+++	–	–	–	/n.a.	+
Subiculum	SUB	S2	+++	–	–	–	/n.a.	+
**Cortical subplate:**		S2						
Claustrum	CLA	S2	++	–	–	–	1/ n.a.	+
Endopiriform nucleus	EP	S2	++	–	–	–	1/ n.a.	+
Amygdala	AMG	S2	++	–	–	–	1/1	+
**Striatum**		S2						
Caudoputamen	CP	S2, S3	–	++	–/+	–	1/1, 2	n.a.
Accumbens	ACB	S2, S3	++	–	+	–	1/2	+
Olfactory tubercle	OT	S2, S3	+++	–	++	–	1/	+
Lateral septal nucleus	LS	S2	+++	–	–	–	/n.a.	+
**Pallidum**		S2					1/2	
Substantia innominata	SI	S2, S3	++	–	+	–		+
Medial septal complex	MSC	S2, S3	+	–	++	–	1/2	n.i.
**Thalamus**							1/n.a.	
Lateral posterior nucleus of the thalamus	LP	S2	++	+	–	–	/n.a.	+
Reticular nucleus thalamus	RT	S2	+++	–	–	–	/n.a.	+
Ventral geniculate nucleus	GENv	S2	+	–	–	–	/n.a.	+
Medial habenula	MH	S2	+++	–	–	–	/n.a.	+
Lateral habenula	LH	S2	+	–	–	–	/n.a.	n.i.
**Hypothalamus**		S2					1/2	
Medial mammillary nucleus	MM	S2	–	+	–	–	n.a./n.a.	n.a.
Posterior hypothalamic nucleus	PH	S2	++	–	–	–	/n.a.	+
Arcuate hypothalamic nucleus	ARH	6C, S2	++	–	–	–	/n.a.	+
Dorsomedial hypothalamic nucleus dorsal part	DMH	S2	+	–	–	–	/n.a.	+
Preoptic area	PO	6A, S2	++	–	–	–	/n.a.	+
Anterior hypothalamic area	AH	6F, S2, S3	–	–	++	–	n.a./	n.a.
Lateral hypothalamic area	LHA	6B, S2	++	–	–	–	/n.a.	+
Zona incerta	ZI	S2, S3	++	–	–/++	–		+
**Midbrain**								
Superior Colliculus	SC	S2, S3	++	+	–/+	–		+
Oculomotor nucleus	III	13A, S2,	++	–	++/+	–	/2	+

		S3						
Cuneiform nucleus	CUN	6E, 13B, S2	++	–	+/–	–		+
Midbrain reticular nucleus	MRN	S2	++	–	++/–	–	/2	+
Ventral tegmental area	VTA	S2	++	–	–	–	/n.a.	+
Periaqueductal gray	PAG	S2, S3	++	+	–/+	–		+
Substantia nigra							1/n.a.	
– Compact part	SNc	S2	++	–	–	–	/n.a.	+
– Reticular part	SNr	S2	++	–	–	–	/n.a.	+
Anterior pretectal nucleus	APN	S2	+	–	–	–	1/n.a.	+
Nucleus of the Optic tract	NOT	S2	+	–	–	–	/n.a.	+
Pedunculopontine nucleus	PPT	S2	+	–	+/–	–		
Interpeduncular nucleus	IPN	10C, S2	–	+++	+/–	–	n.a./2	+
Dorsal raphe nuclei	DR	S2	++	+	–	–	/n.a.	+
**Hindbrain**								
Lateral lemniscus nucleus	NLL	6D, S2	+	–	–	–	/n.a.	n.i.
Medial paralemniscal nucleus	PLN	6D, S2	+	–	+/–	–		n.i.
Principal sensory nucleus of the trigeminal	PSV	S2	++	–	–	–	/n.a.	+
Parabrachial nucleus	PB	S2	+	–	–	–	/n.a.	+
Olivary Complex	SOC	S2	+	–	–	–	/n.a.	+
V motor nucleus of trigeminal	V	14B, S2	+++	–	++/–	–	/2	+
Locus ceruleus	LC	13C, S2	++	–	++	–	/2	+
**Medulla**								
VII Facial motor nucleus	VII	6E, S2	++	–	–	–	/n.a.	+
**Fiber tracts**								
Olfactory nerve layer	onl	S2	–	++	+	+	n.a./	n.a.
Internal capsule	int	S2	–	–	–	n.a./+	n.a./n.a.	n.a.
Fasciculus retroflexus	fr	S2	–	+++	–	–	n.a./n.a.	n.a.
Fimbria	fi	S2	–	++	–	–	n.a./n.a.	n.a.
**Others**								
Subependymal zone	SEZ	S2	++	–	–	–	/n.a.	–
Subcommisural organ	SCO	S2, S3	+++	–	++	–		n.i.
